# Durable Interactions of T Cells with T Cell Receptor Stimuli in the Absence of a Stable Immunological Synapse

**DOI:** 10.1016/j.celrep.2017.12.052

**Published:** 2018-01-09

**Authors:** Viveka Mayya, Edward Judokusumo, Enas Abu Shah, Christopher G. Peel, Willie Neiswanger, David Depoil, David A. Blair, Chris H. Wiggins, Lance C. Kam, Michael L. Dustin

**Affiliations:** 1 Kennedy Institute of Rheumatology, University of Oxford, Oxford OX3 7FY, UK; 2 Skirball Institute of Biomolecular Medicine, New York University School of Medicine, New York, NY 10016, USA; 3 Department of Biological Engineering, Columbia University, New York, NY 10027, USA; 4 Machine Learning Department, Carnegie Mellon University, Pittsburgh, PA 15213, USA; 5 Department of Applied Physics and Applied Mathematics, Columbia University, New York, NY 10027, USA

**Keywords:** lymphocytes, migration, immunological synapse, kinapse, chemokines, adhesion, live imaging, micro-contact printing

## Abstract

T cells engage in two modes of interaction with antigen-presenting surfaces: stable synapses and motile kinapses. Although it is surmised that durable interactions of T cells with antigen-presenting cells involve synapses, *in situ* 3D imaging cannot resolve the mode of interaction. We have established *in vitro* 2D platforms and quantitative metrics to determine cell-intrinsic modes of interaction when T cells are faced with spatially continuous or restricted stimulation. All major resting human T cell subsets, except memory CD8 T cells, spend more time in the kinapse mode on continuous stimulatory surfaces. Surprisingly, we did not observe any concordant relationship between the mode and durability of interaction on cell-sized stimulatory spots. Naive CD8 T cells maintain kinapses for more than 3 hr before leaving stimulatory spots, whereas their memory counterparts maintain synapses for only an hour before leaving. Thus, durable interactions do not require stable synapses.

## Highlights

• Naive T cells spend more time in the motile kinapse state
• Only human memory CD8 T cells spend more time in the stable synapse state
• Kinapses do not reduce durability of interaction with cell-sized stimulatory spots
• Spatial restriction of TCR stimulation does not force formation of a synapse

## Graphical Abstract

**Figure:**
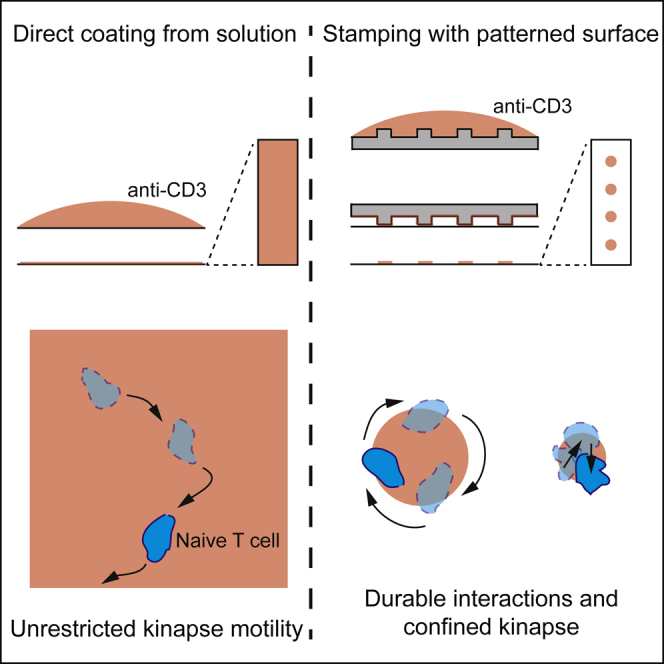

## Teaser

T cells primarily form two types of adhesive junctions when interacting with stimulatory surfaces: stable synapses and motile kinapses. Mayya et al. demonstrate that durable interactions with antigen do not require formation of a stable synapse.

## Introduction

T cell priming requires hours of interaction with cognate antigen-presenting cells (APCs) (Iezzi et al., 1998). Results from *in vivo* imaging suggest that T cells decelerate and arrest on APCs to achieve this duration of signaling when antigen is spatially limited (Mempel et al., 2004, Miller et al., 2004) but can continue to migrate throughout the APC network when antigen is present on many contiguous APCs (Friedman et al., 2010, Hugues et al., 2004, Moreau et al., 2012, Sims et al., 2007). *In vitro* studies indicate two modes of interaction of T cells with APCs that may account for these observations: symmetric and stable synapses and asymmetric and motile kinapses (Dustin, 2007, Friedman et al., 2010, Sims et al., 2007). Decelerated movement of T cells *in vivo* within networks of stimulatory APCs arises from kinapses. Generally, durable interactions of T cells *in vivo* with spatially isolated stimulatory APCs are interpreted to arise from synapses. However, whether durable interactions are mediated by synapses or confined kinapses is not ascertainable because of the inability to resolve details of the interface over time. This inability is mainly a result of internal tissue movement and inherent limitations of 3D rendering. Synapses and kinapses have functional implications, with synapses being more efficient for effector functions (Beal et al., 2008, Huse et al., 2006) and kinapses allowing greater exploration of local networks (Moreau et al., 2015). However, it has been proposed that the polarized distribution of the motility apparatus along the plane of contact in the kinapse mode limits the durability of interaction (Davis, 2009, Dustin, 2007, Gunzer et al., 2000, Moreau and Bousso, 2014). Our goal in this study was to establish an *in vitro* platform under optically ideal settings to determine cell-intrinsic modes of interaction of different T cell subsets when faced with continuous or spatially restricted stimulation and to examine the relationship between the mode and durability of interaction.

We have studied the cell-intrinsic behavior of freshly isolated human and mouse T cells using 2D stimulatory surfaces on glass supports because of the ideal optics. Spatially continuous stimulatory surfaces are based on classical coating approaches or supported planar lipid bilayers (SLBs) presenting ICAM1 and anti-CD3 (Dustin et al., 1997, Parsey and Lewis, 1993). Using such 2D substrates, we found that human naive CD8, human naive and memory CD4, and murine naive and memory CD8 T cells all spend more time in the kinapse mode. Only human memory CD8 T cells formed a majority of synapses. To quantify the duration of interaction with spatially limited stimulation, we combined a 2D chemokinetic substrate composed of ICAM1 and CCL21 (Woolf et al., 2007) with discrete spots of anti-CD3 formed by micro-contact printing (Shen et al., 2008b). This system recapitulates the basic features necessary for T cell scanning, deceleration, and durable interactions observed *in vivo*. Surprisingly, we did not observe the expected inverse correlation between kinapses and durability of interaction. Further, we found that kinapse motility is intact on the stimulatory spots even as naive T cells undergo durable interactions over hours and that spatial restriction of anti-CD3 did not force formation of a stable synapse or exit from the anti-CD3 spots. This result demonstrates that naive T cells can achieve durable interactions for priming without forming stable synapses.

## Results

### Migratory Response of T Cell Subsets to TCR Stimulation

**Figure 1:**
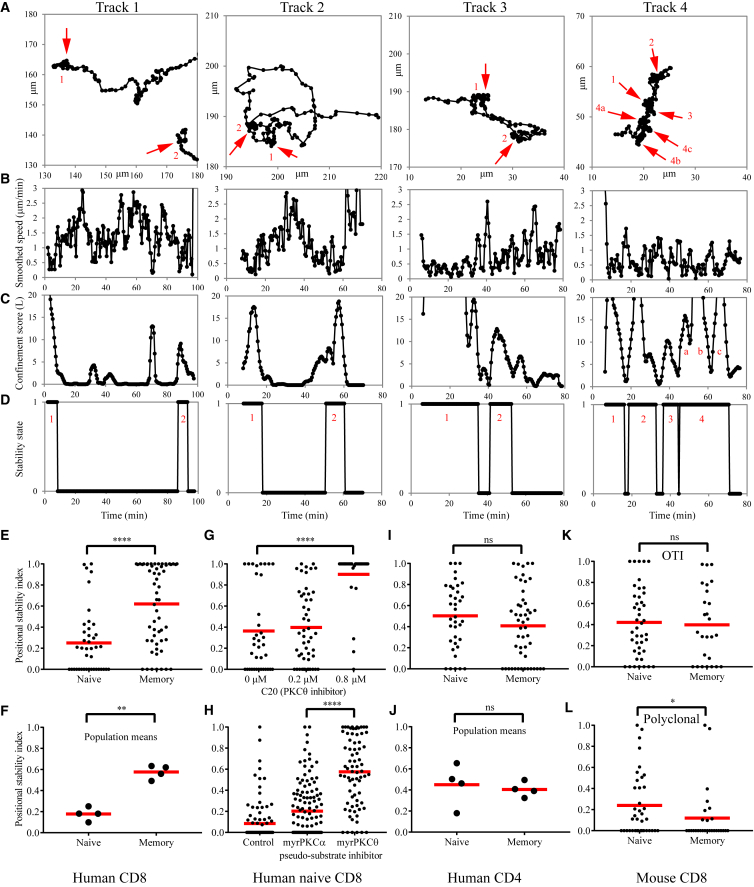
All Major Resting T Cell Subsets, Except Human CD8 Memory Cells, Spend More Time in the Kinapse Mode during Interaction with Uniformly Coated Stimulatory Surfaces (A) Four representative tracks of human naive CD8 T cells. Periods of positional stability, presumably corresponding to synapse mode of interaction, are highlighted using red arrows and numbered for reference. Additional periods of positional stability that one may visually infer are either of too short a duration or represent drastic turns during motility. (B) The speed of the cells shown in (A) over the duration of the tracks. The speed plotted here is smoothed by averaging the instantaneous speed over the two frames before and after the frame in question. However, the profile shows abrupt changes and fails to demarcate the periods of positional stability. (C) The confinement score (originally termed probability level, L) identifies the periods of relative confinement (Simson et al., 1995). L > 3 represents a probability of < 0.017 that the confinement is due to random chance, which is the threshold value used to demarcate periods of relative confinement. (D) The positional spread within each period of relative confinement is considered to determine whether the cell was in the stable state (value of 1) that is synonymous with synapse mode of interaction. Positional spread is defined by R^2^/t, where t is the period of relative confinement, and R is the diameter of the confined zone. A value of < 0.666 μm^2^/frame was found to represent positional stability. The fraction of time the cell spends in the stable state is called the positional stability index. Thus, a positional stability index of > 0.5 means that the cell has spent more time in stable synapse mode than in motile kinapse mode and vice versa. (E–L) The positional stability index of resting T cell subsets on coverglass uniformly coated with anti-CD3 and ICAM1. The type of T cell subset examined is denoted at the bottom or in the panels and in the category names of dot plots (for example, naïve and memory cells from human CD8 T cells in E and human CD4 T cells in I). Inhibitors of PKCθ shift the balance from kinapses toward synapses in human naive CD8 T cells (G and H). The data points in (F) and (J) represent population means from separate blood donors, whereas, in rest of the panels, they represent individual tracks of cells from a particular donor. Mean values are given as red horizontal bars. The data shown in (G), (H), (K), and (L) are representative of two independent experiments.

We first utilized an established 2D T cell migration platform to test the cell-intrinsic tendency of T cell subsets to form synapses and kinapses in response to TCR ligation. Although prior studies suggest that human mixed naive and memory CD4 T cells form kinapses in 2D (Zanin-Zhorov et al., 2010) and 3D (Gunzer et al., 2000) settings, a systematic analysis of naive and memory subsets from peripheral blood has not been undertaken. Glass surfaces were uniformly coated with anti-CD3ε and ICAM1, and the migration of freshly isolated T cells was tracked over a period of 2 hr. Initially we used 2 μg/mL of anti-CD3ε for adsorption, which resulted in a surface density that caused nearly all cells to form adhesive contacts (Movie S1). Human naive CD8 T cells were seen to exhibit periods of positional stability that are synonymous with the stable synapse as well as motility that is characteristic of the kinapse mode (Figure 1A; Movie S1; Sims et al., 2007). To identify these periods of positional stability and motility, we first considered the speed of the cells (Figure 1B). Although the speed was generally reduced during periods of positional stability, it was hard to demarcate these periods with any certainty because of the small range of speed within which the cells move and because of constant, abrupt changes in speed. Therefore, we implemented an algorithmic approach to identify the transient, relative confinement that was originally developed for the analysis of single-particle tracks of membrane proteins (Simson et al., 1995; Figure 1C). We then determined the positional spread during periods of relative confinement to confirm whether the cell was in synapse mode (Figure 1D; Supplemental Experimental Procedures). After ascertaining the mode of interaction at every instance, we calculated the “positional stability index,” which is defined as the fraction of time the cell spends in the stable synapse state. This parameter was used to quantify the intrinsic tendency of the T cells to form synapses instead of kinapses or vice versa.

Human naive CD8 T cells formed kinapses, whereas the memory counterparts spent more time in the synapse mode, based on analysis of individual cells from a single donor (Figure 1E; Movie S1), and when mean values from 4 donors (Figure 1F) were considered. To relate this to prior observations, we investigated the role of protein kinase Cθ (PKCθ), which promotes kinapse motility in murine naive CD4 T cells *in vitro* and *in vivo* (Sims et al., 2007). Small-molecule (Figure 1G) or peptide-based inhibitors (Figure 1H) of PKCθ increased the positional stability index for the naive CD8 T cells. Thus, the motility observed in this model system is analogous to kinapses observed *in vivo* and on SLBs. We expanded our observations to other human and mouse T cells subsets. Naive and memory human CD4 T cells spent more time in the kinapse mode both within a population of cells (Figure 1I) and between 4 donors (Figure 1J). Furthermore, naive and anti-*Listeria* memory CD8 T cells from OT-I TCR transgenic mice (Figure 1K) or polyclonal *Listeria*-specific memory CD8 T cells from B6 mice (Figure 1L) all formed kinapses. OT-I naive T cells are known to form kinapses on SLBs presenting peptide-major histocompatibility complex (pMHC) and ICAM1 and *in vivo* upon intravenous (i.v.) injection of the cognate peptide (Friedman et al., 2010). Furthermore, the distribution of migratory speed of P14 T cell receptor (TCR) transgenic naive and memory T cells in the presence of antigen-loaded dendritic cells (DCs) is also reflective of kinapse motility *in vivo* for both subsets (Sung et al., 2012). Overall, across all populations, only human memory CD8 T cells exhibited an intrinsic tendency to form synapses for a longer duration, whereas the other human and mouse subsets we examined spent more time in the kinapse mode.

**Figure 2:**
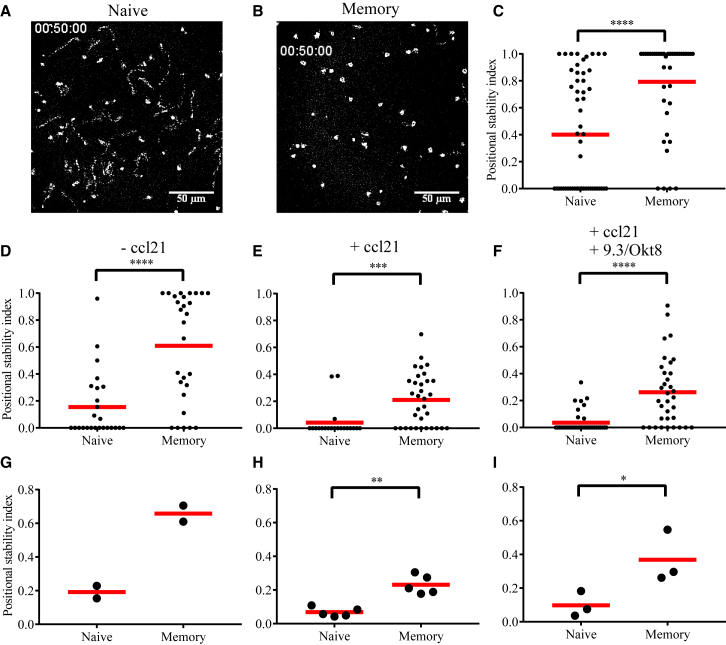
The Lower Positional Stability Index of Human Naive CD8 T Cells Remains Intact Even When the Characteristics of the Stimulating Surface Change (A and B) Micrographs of fluorescent UCHT1 Fab′ taken 50 min after the cells were introduced on SLBs presenting UCHT1 Fab′ and ICAM1 as freely mobile ligands. The images show TCR clusters confined to the interface (B) because of the synapse of memory cells and trail of UCHT1 (A) shed by kinapses of naive cells. The data shown are representative of two independent experiments. (C) Positional stability index of cells quantified from tracks on SLBs. (D–I) Positional stability index of human CD8 T cells on coverglass presenting the threshold density of immobilized OKT3, below which very few naive cells respond by attaching. The presence of additional ligands (CCL21 in E, F, H, and I and 9.3 and OKT8 antibodies in F and I, with no additional ligand in D) is noted at the top. Immobilized ICAM1 was present in all experiments. The data points in (G), (H), and (I) represent population means from separate blood donors, whereas, in the rest of the panels, they represent individual tracks of cells from a particular donor. Mean values of plotted data points are given as red horizontal bars.

We wanted to further investigate the contrasting tendencies of naive and memory human CD8 T cells under different conditions. We first assessed the mode of interaction on SLBs presenting fluorescently labeled anti-CD3 Fab′ and ICAM1. In this model, kinapse motility can be easily identified through the trail of TCR-enriched micro-vesicles shed by migrating cells, whereas cells with stable synapses maintain TCR micro-vesicles within the interaction interface (Choudhuri et al., 2014). Naive human CD8 T cells spent more time in the kinapse mode, and memory CD8 T cells predominantly formed synapses on SLBs (Figures 2A–2C). This was found to be the case even at the drastically reduced density of anti-CD3 adsorbed to glass (Figures 2D and 2G). Although the homeostatic lymphoid chemokine CCL21 reduces the time T cells spend in synapse mode, memory CD8 T cells consistently spend more time in synapse mode than naive cells (Figures 2E, 2F, 2H, and 2I). The behavior of naive cells did not change appreciably when costimulatory and co-receptors were also engaged using antibodies (Figures 2F and 2I). Together, we conclude that, with the exception of human memory CD8 T cells, all other resting T cell subsets we examined have a cell-intrinsic tendency to predominantly form kinapses on uniform stimulatory surfaces.

### Durability of Interaction on Stimulatory Spots

**Figure 3:**
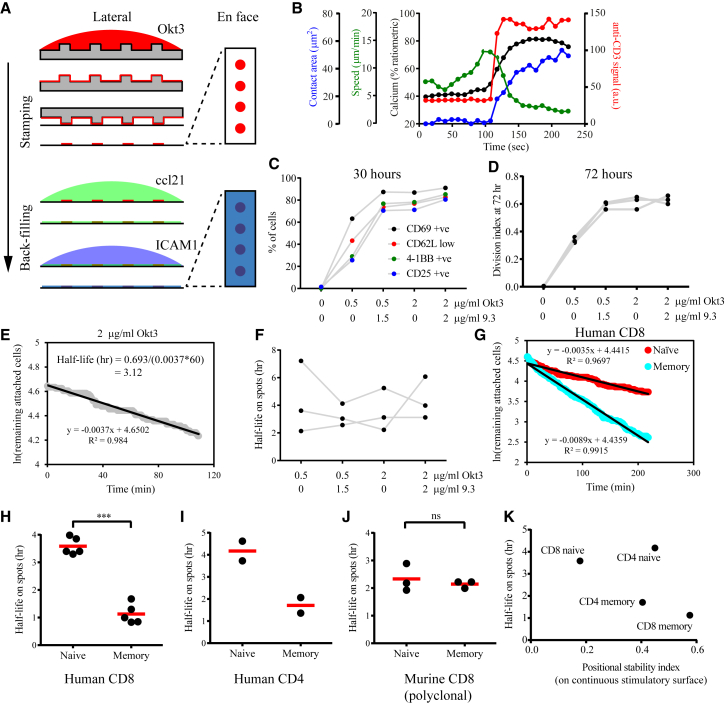
The Durability of Interaction of Human T Cell Subsets with Spatially Limiting Stimulatory Spots Does Not Correlate with the Arrest Coefficient (A) Schematic of the micro-contact printing procedure for making stimulatory spots that emulate the spatially limiting and distributed nature of antigen presentation in lymph nodes with pervasive adhesion ligands and homeostatic chemokines. Anti-CD3ε is adsorbed on to the polydimethylsiloxane (PDMS) cast with patterned indentations. The PDMS is then stamped to transfer some of the adsorbed protein onto the coverglass. The entire surface is then coated sequentially with the chemokine CCL21 and ICAM1. See Experimental Procedures for details. (B) Calcium influx (in black), arrest (in green), and spreading (in blue) of motile human naive CD8 T cells on the stimulatory spots (in red) captured by temporally aligning tracks of cells as they reach and arrest on the spots. Average values from 20 cells based on such a virtual synchronization are shown. (C) Activation status of cells after 30 hr of interaction with the stimulatory spots presenting varying amounts of Okt3 and 9.3 antibodies. See also Figure S1. (D) Proliferation of naive CD8 T cells from 3 donors after 72 hr of interaction with the stimulatory spots presenting varying amounts of Okt3 and 9.3 antibodies. The division index is the average number of divisions for all cells present. (E) Illustration for the calculation of the half-life of interaction with stimulatory spots. T cells are introduced to find and arrest onto the spots. Live-cell imaging commences when at least ∼50% of the spots are occupied by arrested cells. The cells found to be initially arrested on the spots are tracked through the time lapse, and the percentage of cells remaining on the same spots is tallied. Natural logarithmic transformation of the percentage of remaining cells provides a curve with a very good linear fit. The half-life of interaction is calculated by the formula ln(2)/slope according to first-order kinetics. (F) Co-stimulation by the 9.3 antibody did not influence the half-life of interaction with stimulatory spots. (G) A larger percentage of memory CD8 T cells (in cyan) leave the spot onto which they had initially arrested, reflecting a shorter half-life of interaction. It is to be noted that memory cells, or, for that matter, naive cells, that leave a spot typically engage and attach onto another neighboring spot. However, such re-engagements are disregarded in this “survival” analysis. (H–J) Half-life of interaction of specified T cell subsets upon attachment. The type of T cell subset examined is denoted at the bottom and in the category names of the dot plots (human CD8s in H, human CD4s in I, and murine CD8s in J). Human naive T cells have an appreciably longer half-life of interaction compared with the memory counterparts. Mean values of plotted data points are given as red horizontal bars. Each half-life measurement shown here came from different donors. (K) Relationship between the half-life of interaction on stimulatory spots and the positional stability index on a continuous stimulatory surface among human T cell subsets. Mean values from all the donors examined are plotted.

We then tested whether kinapse-based motility leads to a reduced duration of interaction on spatially limiting and distributed stimulatory spots created by micro-contact printing (Figure 3A). Patterned surfaces have previously been used to explore the influence of different spatial patterns of ligands on the extent of T cell activation (Doh and Irvine, 2006, Shen et al., 2008b). In our case, the micro-patterned antigen-presenting surface was particularly inspired by and designed to emulate the spacing of individual antigen-loaded DCs within the network of DCs in lymph nodes (Lindquist et al., 2004). Micro-patterned printing of anti-CD3 provides stimulatory spots with biophysical and biochemical characteristics very similar to uniform stimulatory surfaces. Thus, it is expected that both initiate the same mode of triggering of TCRs and allow for direct comparison of results from the two model stimulatory surfaces. An additional critical feature of the T cell area in the lymph node is the presentation of the homeostatic chemokine CCL21 by stromal cells and lymphoid-resident DCs, which prompts the scanning motility of naive T cells (Bousso, 2008). Glass-adsorbed CCL21 has been shown to prompt a similar persistent motility in T cells (Woolf et al., 2007). Adoption of this approach with micro-contact printing allows the T cells to efficiently locate and interact and engage with the stimulatory spots and mimic the main aspects of the *in situ* environment in which naive T cells are primed.

Human naive CD8 T cells scanned the surface, decelerated, arrested, and attached to the 10-μm stimulatory spots placed 30 μm apart on a square grid (Figure 3B; Movie S2, bottom left quadrant). Robust intracellular calcium flux was also observed as the cells arrested and spread, indicating productive supra-threshold TCR signaling. We then asked whether the naive T cells are indeed primed on the stimulatory spots. 50% of the cells were found to have expressed CD69 and shed CD62L after 12 hr (Figure S1A). By 30 hr, 80% of the cells express CD69, along with other activation markers such as CD25 and 4-1BB (Figure 3C; Figure S1G). By 72 hr, the cells had divided at least twice (Figure S1F). Thus, anti-CD3 alone on the stimulatory spots is sufficient to prime naive CD8 T cells. We assessed the influence of co-stimulation by stamping the 9.3 antibody clone against CD28 along with Okt3. A robust effect of co-stimulation was observed only when 0.5 μg/mL of Okt3, but not 2 μg/mL of Okt3, was used for stamping (Figures 3C and 3D; Figures S1B–S1I). Also, increasing the concentration of Okt3 from 0.5 to 2 μg/mL obviated the need for co-stimulation during priming. By following the fate of attached cells regarding when they exit the stimulatory spots, we can calculate the half-life of interaction (Figure 3E). Surprisingly, we did not observe any decisive contribution of co-stimulation toward the durability of interaction with the stimulatory spots (Figure 3F). Therefore, for all ensuing experiments comparing naive and memory T cells, we considered anti-CD3 alone (at 2 μg/mL) on the stimulatory spots.

We found that the human naive CD8 T cells exited the spots at a slower rate than memory CD8 T cells (Figure 3G; Movie S2, top half). Human naive CD8 T cells were found to interact with a half-life of 3.6 hr, whereas the memory CD8 T cells had a half-life of 1.1 hr on 10-μm spots (Figure 3H). Similar results were obtained in the human CD4 subsets (Figure 3I). Both human CD4 and CD8 memory cells have less durable interactions on the stimulatory spots. Both of these subsets are known to have reduced phosphorylation of TCR-proximal signaling proteins and reduced calcium levels (Adachi and Davis, 2011). Accordingly, we observed reduced calcium influx in memory CD8 T cells on the stimulatory spots (Figure S1J). This offers a likely explanation for the less durable interaction of memory cells. Murine polyclonal CD8 naive and anti-*Listeria* memory T cells were found to have an intermediate half-life of ∼2 hr on 10-μm spots (Figure 3J). This result is consistent with *in vivo* observations wherein adoptively transferred naive and *ex vivo*-generated memory P14 TCR transgenic CD8 T cells engaging with lipopolysaccharide (LPS)-activated DCs that migrate from the footpad into the popliteal lymph node were found to have the same contact duration with the DCs (Sung et al., 2012). Overall, we did not observe the expected positive correlation between positional stability index on uniform stimulatory surfaces and durability of interaction on stimulatory spots (Figure 3K).

### The Motile Tendency of Kinapses Is Intact on Stimulatory Spots

**Figure 4:**
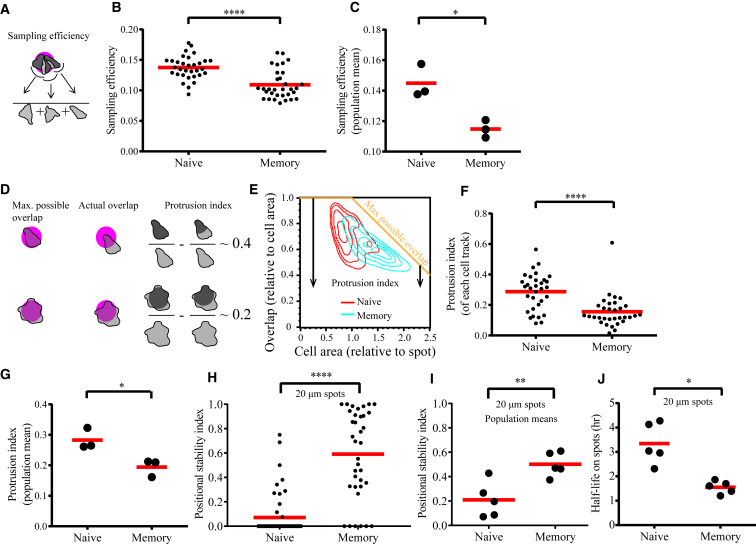
Human Naive CD8 T Cells Exhibit Durable Interactions Despite Kinapses on the Stimulatory Spots (A–G) The motile tendency of human CD8 T cells engaged on 10-μm spots is quantified as sampling efficiency (A–C) and protrusion index (D–G). (A) Sampling efficiency is defined as the fraction of unique pixels (denoted by darker shade of gray) over the total number of pixels (denoted by lighter shade of gray in the denominator) underneath the cell boundary within a certain duration of time (the illustration has 3 time steps). A cell with increased motile tendency should have a higher sampling efficiency. (B and C) Naive cells have a higher sampling efficiency than memory cells when measured over 20 time steps. The same conclusion is drawn when 10 or 40 time steps were considered (data not shown). See also Figure S2. (D) The protrusion index measures the fractional area of the cell that is outside of the stimulatory spot, which should increase with a more active search away from the stimulatory spot. The protrusion index of a cell at any instance is calculated by subtracting the fractional overlap from the maximum possible overlap (overlap is shown in dark gray). The maximum possible overlap reduces as the projected area of the cell increases beyond the size of the spot. Thus, considering the maximum possible overlap accounts for the increase in protrusion solely because of increased size and/or spreading of the cell. (E) Contour map of the fractional overlap of naive (in red) and memory (in cyan) CD8 T cells. The contour plot was generated from data points representing each cell at each time point imaged. As the area of the cell grows beyond the area of the spot, the maximum possible overlap decreases linearly (orange trace). For any cell represented in the plot, the distance from the orange trace gives the protrusion index (shown by arrows for two cells). (F and G) Naive cells have a higher protrusion index than memory cells. (H and I) Naive human CD8 T cells exhibit a greater tendency for kinapses on 20-μm spots compared with memory cells. Note that only portions of tracks that did not contain any neighboring cell on the same spot were considered for this analysis to select for autonomous behavior. (J) Human naive CD8 T cells exhibit a longer half-life of interaction on 20-μm-wide spots. This was the case even when the spots were completely occupied by crowding with 4–6 cells per spot instead of 1–3 cells per spot (data not shown). The data points in (B), (F), and (H) represent individual cells or cell tracks from a particular donor, and those in (C), (G), and (I) represent population means from separate blood donors. Mean values of plotted data points are given as red horizontal bars.

We considered the possibility that the behavior of naive cells is fundamentally different on the spatially confined stimulatory spots, which might force formation of synapses. For this, we focused on comparison of human naive and memory CD8 T cells because they had exhibited a stark dichotomy in behavior (Figures 1 and 3). Visual examination of the dynamics of naive cells engaged on 10-μm-wide stimulatory spots revealed that the motile tendency generated by kinapses is intact and that the cells continuously explore new areas (Movie S3, for example). To quantify this, we defined a “sampling efficiency” parameter that measures the fraction of unique pixels within the cell outline over 20 frames or 10 min (Figure 4A). Naive cells exhibited a significantly greater sampling efficiency than the memory counterparts on the spots (Figures 4B and 4C), as observed on the uniformly coated surfaces (Figures S2A and S2B). We found that naive cells displayed significantly greater sampling over a range of frame numbers and intervals (data not shown). We also noted that the naive cells displayed continuous protrusions in different directions away from the spot; however, they still remained on the spot, apparently because of interspersed preferential retraction from the non-stimulatory area (Movie S3). To quantify this behavior, we defined a “protrusion index” parameter that measures the fractional area of the cell that is outside of the spot, taking into account variations in the projected area of the cell relative to the size of the spot (Figures 4D and 4E). The naive cells exhibited a significantly higher protrusion index than memory cells (Figures 4F and 4G). Thus, quantification of the motile tendencies in human naive and memory CD8 T cells revealed that the intrinsic behavior observed on uniform stimulatory surfaces is preserved on 10-μm stimulatory spots. Dynamic sampling and protrusive behavior were also observed in all the other resting T cell subsets examined, suggesting that they all retained their kinapse tendencies on 10-μm spots (data not shown).

Human memory CD8 T cells form larger contacts on uniformly coated surfaces (Movie S1) and on 10-μm-wide spots (Figures 4E; Figure S2C). The memory cells also have a lesser portion of the cell surface exposed to the stimulatory spots (Figure S2D). Thus, it is possible that memory cells actively decide to dissolve the synapse on account of sub-optimal surface exposure on 10-μm spots, leading to under-estimation of the potential for durable interactions. Therefore, we extended the comparisons to 20-μm stimulatory spots. Many human naive CD8 T cells were seen to circle along the edges on the 20-μm-wide spots because their persistent movement always led them to form and retract protrusions off of the spot (Movie S4, for example). Overall, the naive cells showed a significantly lower positional stability index on 20-μm spots compared with the memory cells (Figures 4H and 4I). This was directly analogous to the behavior on uniformly coated surfaces (Figures 1E and 1F and 2E and 2H). The naive cells maintained the trend of a longer half-life of interaction (an average of 3.4 hr) despite continuous motility (Figure 4J). Further, human naive CD8 T cells exhibited durable interactions with mature monocyte-derived DCs embedded in collagen matrix and presenting Okt3 that was captured via their Fc receptors (Movies S5 and S6). During these prolonged interactions, the naive cells exhibit continuous protrusions in different directions. One such event led to the naive cell shifting from one DC to another DC that comes into the neighborhood (Movie S5). This provides further proof that kinapse motility is intact during the prolonged interaction with DCs. Thus, our results demonstrate that naive T cells have prolonged interactions without forming a stable synapse and despite motile tendencies driven by the kinapse. We conclude that kinapses are not detrimental to the durability of interaction with spatially restricted stimulatory sites or with DCs.

## Discussion

Our *ex vivo* experiments mimic the two distinct scenarios of antigen presentation resulting from standard immunization regimens and allow the interaction mode to be determined quantitatively. Uniformly coated surfaces and SLBs emulate the scenario arising after peptide- or antigen-conjugated DEC-205 immunization, which results in spatially uniform antigen presentation by the DC network (Friedman et al., 2010, Hugues et al., 2004, Moreau et al., 2012, Sims et al., 2007). Kinapse motility observed *in situ* does not become a detrimental factor for prolonged signaling under these conditions because T cells remain in contact with antigen even as they move from DC to DC. Micro-patterned surfaces mirror the scenario arising after DC immunization, in which antigen is presented in a spatially restricted manner by the emigrated DCs within the T cell zone. Taking advantage of the unique settings of optics and the substrate design in 2D, we have demonstrated that kinapse motility is intact on the stimulatory spots (Figure 4; Movies S3 and S4). However, these T cells exhibit durable interactions (Figures 3H and 3I and 4J), just as *in vivo* with DC immunization (Celli et al., 2007, Henrickson et al., 2008, Mempel et al., 2004). Motile tendency and crawling, similar to what we have observed on 10- and 20-μm stimulatory spots, can also be gleaned from the time-lapse images of T cell-dendritic cell interaction (T-DC) conjugates *in situ* (Figure S2E lists specific instances from the literature). This implies that the mode of engagement in those instances was via kinapses and not synapses. Thus, the extent of spatial distribution of antigen or anti-CD3ε dictates the spatial regime of naive T cell motility over several hours, either restricted to a single DC (or stimulatory spot) or across a larger area. This allows for reconciliation between the two seemingly incompatible features of T cell behavior: kinapse motility and durable interaction with DCs, which is required for the priming of naive T cells. We further note that our results do not rule out durable interactions in the synapse mode of interaction. Thus, durable interactions could arise both from synapses and kinapses.

Human memory T cells demonstrate a trend of lower half-life of interaction compared with their naive counterparts (Figures 3 and 4). We note that, after the memory cells leave a stimulatory spot, they latch onto another. Thus, the cooperative killing observed in antiviral responses could be a result of kinapses or serial, relatively short synapses (Halle et al., 2016). A shorter duration would allow the higher effector efficiency of synapses to be exploited without completely losing the possibility for local exploration inherent to kinapses (Beal et al., 2008, Huse et al., 2006). This is perhaps the reason for human memory CD8 T cells to employ stable synapses but limit their duration.

Our model system of stimulatory spots enabled us to demonstrate that T cells can engage in durable interactions without forming stable synapses. Because this model system mimics the main aspects of the *in situ* context under which naive T cells get primed (Bousso, 2008, Lindquist et al., 2004), we expect it to be of great utility in investigating the mechanistic underpinnings of T cell behavior varying from search for antigen and signal integration during priming to competition between T cells for antigen.

## Experimental Procedures

### Ethics

Leukapheresis products (non-clinical and de-identified) from donor blood were used as a source of resting human T cells, which was exempt from institutional review board (IRB) review. The Non-Clinical Issue division of National Health Service approved the use of leukapheresis products at the University of Oxford (REC 11/H0711/7). All procedures and experiments involving mice were conducted at the New York University Medical Center and were approved by the Institutional Animal Care and Use Committee (protocol 150609-01).

### Isolation of T Cell Subsets

Resting human T cell subsets were isolated from leukapheresis products using negative selection kits from STEMCELL Technologies. Mouse T cells were isolated either by negative selection or by sorting of relevant populations from B6 mice. Memory T cells were obtained from mice 30 days after infection with *Listeria monocytogenes*-expressing ovalbumin. OT-I T cells were isolated after adoptive transfer into congenic hosts.

### Preparation of Stimulatory Surfaces

Labtek 8-well chambers (Nunc) were used for uniformly coated stimulatory surfaces. Micro-contact printing was carried out as described previously (Shen et al., 2008a). The repeating “spot” patterns spanned the entire length of the channel of the sticky-Slide VI^0.4^ (Ibidi). The stamped coverslips were affixed to the sticky-Slide, and the channels were coated sequentially with CCL21 (10 μg/mL) and ICAM1 (3 μg/mL). SLBs presenting UCHT1 Fab′ and ICAM1 were assembled in sticky-Slide VI^0.4^ channels essentially with the same approach as described before for the FSC2 Bioptechs flow chambers (Choudhuri et al., 2014, Dustin et al., 2007). The main difference is that the entire channel was filled with the liposome suspension to form a bilayer all along the channel.

### Imaging

Cells were imaged using either a Zeiss LSM 510 or an Olympus FluoView FV1200 confocal microscope that was enclosed in an environment chamber (at 37°C) and operating under standard settings. These microscopes are equipped for collecting differential interference contrast (DIC) images used for detecting and tracking cells and for collecting reflection images used for ascertaining spreading or attachment. In some experiments, cells are labeled with CellTracker dyes (Life Technologies) to identify the T cells subsets. Calcium was imaged by the ratiometric method using Fluo4-acetylmethoxy ester. (AM) and Fura Red-AM (Wolf et al., 2015). The location of stimulatory spots was recorded using Alexa Fluor 647 conjugated to the stamped anti-CD3.

### Image Analysis and Quantification of Various Metrics

The time-lapse images were pre-processed in ImageJ. Tracking and associated quantification were conducted using TIAM, a MATLAB-based toolset we have developed (Mayya et al., 2015). The code is available on Github (https://github.com/willieneis/TIAM). Bespoke functions and scripts were written in MATLAB for the calculation confinement score, positional stability index, sampling efficiency, protrusion index, and duration of interaction, with the spots using the output from TIAM. These functions are scripts also available on Github (https://github.com/uvmayya/kinapseVsDurability).

### T Cell Activation Assays

Cell numbers equivalent to the number of stimulatory spots (∼90,000) were introduced into the channel. The wells feeding the channel were simultaneously filled with additional medium. At various time points, the cells were collected using ice-cold PBS containing 0.5% BSA and 2 mM EDTA. These cells were appropriately assayed by flow cytometry for activation markers and cell division by dilution of Cell Trace Violet.

### Statistical Methods

Statistical significance of difference in values, wherein a pair of values represents the T cell subsets of a donor, was calculated by paired t test. Statistical significance of difference in population behavior, wherein individual cells between subsets of a donor are compared, was calculated by Mann-Whitney *U* test. p values from two-tailed tests are denoted as follows in the figures: ^∗^p < 0.05, ^∗∗^p < 0.01, ^∗∗∗^p < 0.001, ^∗∗∗∗^p < 0.0001.

All experimental and analysis procedures are explained in further detail in the Supplemental Experimental Procedures.

## Supplemental Information

Document S1. Supplemental Experimental Procedures, Figures S1–S6, Tables

Movie S1. Behavior of Human CD8 T Cells on Uniform Stimulatory Surfaces, Related to Figure 1 Time-lapse video of the motility behaviour of CMRA-labelled naïve (in red) and CMFDA-labelled memory (in green) human CD8 T cells on immobilized OKT3 and ICAM1. Field of imaging was 131.84 μm in size. Progression of time is also noted in the video.

Movie S2. Behavior of Human CD8 T Cells on Cell-Sized Stimulatory Spots, Related to Figure 3 Side-by-side comparison of durability of interaction of naïve (on left) and memory (on right) human CD8 T cells with 10μm stimulatory spots. Spots are shown in magenta. In the upper half, a positive mask of the dilated spot and contact foot-print was applied to the DIC images so that only the cells engaged on the spots were tracked by TIAM. Masked DIC images used for the video here helps with avoiding distraction from motile cells that pass by the spots. In the lower half, the same dataset is shown without the mask. There was some advection in the channel containing naïve cells, as a results of which motile cells have a bias in their movement towards the top of the field. Nonetheless, scanning motility of T cells can be appreciated in the lower half of the video. It is to be noted that advection does not impact durability of interaction. Tracked positions are overlaid as small yellow squares on the masked DIC images. A bigger yellow square flashes to indicate the termination of the track, in other words, signifies dissolution of the synapse and exit from the spot. Memory cells exhibit more such events, implying reduced half-life of interaction on antigenic spots. There were some technical challenges in calculating the half-life of interaction for memory cells, which are detailed in the Image Analysis sub-section of Supplemental Experimental Procedures. The memory cells that leave a spot, typically engage with another neighboring spot. This can be appreciated by the sizeable number of attached memory cells on spots that were not tracked, as their tracks did not begin at the initially considered frame.

Movie S3. Kinapse Behavior Highlighted for a Single Naïve CD8 T Cell on a 10-μm Spot, Related to Figure 4 Time-lapse video of a single naïve CD8 T cell showing prolonged engagement (3 hours) with a 10μm OKT3 spot despite constant generation of protrusions away from the spot. Occasionally, the cell also forms a nascent uropod and a dominant, single protrusion at the opposite end, both of which are transient. The cell is shown as an overlay of DIC and IRM to provide a darker contrast and thus distinguish the concerned cell from other passing-by cells that transiently engage the spot without a juxtaposed contact. Outline of the spot and the cell boundary are also provided on the right side to aid visualization.

Movie S4. Kinapse Behavior Highlighted for a Single Naive CD8 T Cell on a 20-μm Spot, Related to Figure 4 Time-lapse video of a single naïve CD8 T cell showing prolonged engagement (>2 hours) with a 20μm OKT3 spot despite constant kinapse motility along the circumference of the spot. As in Movie S3, the cell is shown as an overlay of DIC and IRM to provide a darker contrast and thus distinguish the concerned cell from other passing-by cells that transiently engage the spot without a juxtaposed contact. Outline of the spot and the cell boundary are also provided on the right side to aid visualization. The motility of the T cell is seemingly confined or dictated by the boundary of the spot.

Movie S5. Naive CD8 T Cells in Prolonged Engagement with DCs while Exhibiting Kinapse Behavior, Related to Figure 4 3D time-lapse video of human naïve CD8 T cells (in green) engaging in prolonged interaction with mature monocyte derived DCs (in magenta) that were loaded with Okt3 via their Fc Receptors and embedded in collagen matrix. Motile tendency and protrusive behaviour persists throughout the 1.5 hours of engagement that was captured. We did not observe naïve cells disengaging from the DCs within the 1.5 hours of observation. The migratory movement of other cells in the field was primarily due to CCL19 that was added to the collagen matrix. The engaged T cell shifts from one DC to another DC that comes into the vicinity. This implies kinapse mode of interaction with the DCs.

Movie S6. Naive CD8 T Cells in Prolonged Engagement with DCs while Exhibiting Kinapse Behavior, Related to Figure 4 3D time-lapse video of human naïve CD8 T cells (in green) engaging in prolonged interaction with mature monocyte derived DCs (in magenta) that were loaded with Okt3 via their Fc Receptors and embedded in collagen matrix. Motile tendency and protrusive behaviour persists throughout the 1.5 hours of engagement that was captured. We did not observe naïve cells disengaging from the DCs within the 1.5 hours of observation. The migratory movement of other cells in the field was primarily due to CCL19 that was added to the collagen matrix. Multiple naïve cells can be seen jostling with motile tendency, yet they stay engaged with the DC. Observations are representative of two separate experiments.

Document S2. Article plus Supplemental Information

## References

[bib1] Adachi K., Davis M.M. (2011). T-cell receptor ligation induces distinct signaling pathways in naive vs. antigen-experienced T cells. Proc. Natl. Acad. Sci. USA.

[bib2] Beal A.M., Anikeeva N., Varma R., Cameron T.O., Norris P.J., Dustin M.L., Sykulev Y. (2008). Protein kinase C theta regulates stability of the peripheral adhesion ring junction and contributes to the sensitivity of target cell lysis by CTL. J. Immunol..

[bib3] Bousso P. (2008). T-cell activation by dendritic cells in the lymph node: lessons from the movies. Nat. Rev. Immunol..

[bib4] Celli S., Lemaître F., Bousso P. (2007). Real-time manipulation of T cell-dendritic cell interactions in vivo reveals the importance of prolonged contacts for CD4+ T cell activation. Immunity.

[bib5] Choudhuri K., Llodrá J., Roth E.W., Tsai J., Gordo S., Wucherpfennig K.W., Kam L.C., Stokes D.L., Dustin M.L. (2014). Polarized release of T-cell-receptor-enriched microvesicles at the immunological synapse. Nature.

[bib6] Davis D.M. (2009). Mechanisms and functions for the duration of intercellular contacts made by lymphocytes. Nat. Rev. Immunol..

[bib7] Doh J., Irvine D.J. (2006). Immunological synapse arrays: patterned protein surfaces that modulate immunological synapse structure formation in T cells. Proc. Natl. Acad. Sci. USA.

[bib8] Dustin M.L. (2007). Cell adhesion molecules and actin cytoskeleton at immune synapses and kinapses. Curr. Opin. Cell Biol..

[bib9] Dustin M.L., Bromley S.K., Kan Z., Peterson D.A., Unanue E.R. (1997). Antigen receptor engagement delivers a stop signal to migrating T lymphocytes. Proc. Natl. Acad. Sci. USA.

[bib10] Dustin M.L., Starr T., Varma R., Thomas V.K. (2007). Supported planar bilayers for study of the immunological synapse. Curr. Protoc. Immunol..

[bib11] Friedman R.S., Beemiller P., Sorensen C.M., Jacobelli J., Krummel M.F. (2010). Real-time analysis of T cell receptors in naive cells in vitro and in vivo reveals flexibility in synapse and signaling dynamics. J. Exp. Med..

[bib12] Gunzer M., Schäfer A., Borgmann S., Grabbe S., Zänker K.S., Bröcker E.B., Kämpgen E., Friedl P. (2000). Antigen presentation in extracellular matrix: interactions of T cells with dendritic cells are dynamic, short lived, and sequential. Immunity.

[bib13] Halle S., Keyser K.A., Stahl F.R., Busche A., Marquardt A., Zheng X., Galla M., Heissmeyer V., Heller K., Boelter J. (2016). In Vivo Killing Capacity of Cytotoxic T Cells Is Limited and Involves Dynamic Interactions and T Cell Cooperativity. Immunity.

[bib14] Henrickson S.E., Mempel T.R., Mazo I.B., Liu B., Artyomov M.N., Zheng H., Peixoto A., Flynn M.P., Senman B., Junt T. (2008). T cell sensing of antigen dose governs interactive behavior with dendritic cells and sets a threshold for T cell activation. Nat. Immunol..

[bib15] Hugues S., Fetler L., Bonifaz L., Helft J., Amblard F., Amigorena S. (2004). Distinct T cell dynamics in lymph nodes during the induction of tolerance and immunity. Nat. Immunol..

[bib16] Huse M., Lillemeier B.F., Kuhns M.S., Chen D.S., Davis M.M. (2006). T cells use two directionally distinct pathways for cytokine secretion. Nat. Immunol..

[bib17] Iezzi G., Karjalainen K., Lanzavecchia A. (1998). The duration of antigenic stimulation determines the fate of naive and effector T cells. Immunity.

[bib18] Lindquist R.L., Shakhar G., Dudziak D., Wardemann H., Eisenreich T., Dustin M.L., Nussenzweig M.C. (2004). Visualizing dendritic cell networks in vivo. Nat. Immunol..

[bib19] Mayya V., Neiswanger W., Medina R., Wiggins C.H., Dustin M.L. (2015). Integrative analysis of T cell motility from multi-channel microscopy data using TIAM. J. Immunol. Methods.

[bib20] Mempel T.R., Henrickson S.E., Von Andrian U.H. (2004). T-cell priming by dendritic cells in lymph nodes occurs in three distinct phases. Nature.

[bib21] Miller M.J., Safrina O., Parker I., Cahalan M.D. (2004). Imaging the single cell dynamics of CD4+ T cell activation by dendritic cells in lymph nodes. J. Exp. Med..

[bib22] Moreau H.D., Bousso P. (2014). Visualizing how T cells collect activation signals in vivo. Curr. Opin. Immunol..

[bib23] Moreau H.D., Lemaître F., Terriac E., Azar G., Piel M., Lennon-Dumenil A.M., Bousso P. (2012). Dynamic in situ cytometry uncovers T cell receptor signaling during immunological synapses and kinapses in vivo. Immunity.

[bib24] Moreau H.D., Lemaître F., Garrod K.R., Garcia Z., Lennon-Duménil A.M., Bousso P. (2015). Signal strength regulates antigen-mediated T-cell deceleration by distinct mechanisms to promote local exploration or arrest. Proc. Natl. Acad. Sci. USA.

[bib25] Parsey M.V., Lewis G.K. (1993). Actin polymerization and pseudopod reorganization accompany anti-CD3-induced growth arrest in Jurkat T cells. J. Immunol..

[bib26] Shen K., Qi J., Kam L.C. (2008). Microcontact printing of proteins for cell biology. J. Vis. Exp..

[bib27] Shen K., Thomas V.K., Dustin M.L., Kam L.C. (2008). Micropatterning of costimulatory ligands enhances CD4+ T cell function. Proc. Natl. Acad. Sci. USA.

[bib28] Sims T.N., Soos T.J., Xenias H.S., Dubin-Thaler B., Hofman J.M., Waite J.C., Cameron T.O., Thomas V.K., Varma R., Wiggins C.H. (2007). Opposing effects of PKCtheta and WASp on symmetry breaking and relocation of the immunological synapse. Cell.

[bib29] Simson R., Sheets E.D., Jacobson K. (1995). Detection of temporary lateral confinement of membrane proteins using single-particle tracking analysis. Biophys. J..

[bib30] Sung J.H., Zhang H., Moseman E.A., Alvarez D., Iannacone M., Henrickson S.E., de la Torre J.C., Groom J.R., Luster A.D., von Andrian U.H. (2012). Chemokine guidance of central memory T cells is critical for antiviral recall responses in lymph nodes. Cell.

[bib31] Wolf I.M., Diercks B.P., Gattkowski E., Czarniak F., Kempski J., Werner R., Schetelig D., Mittrücker H.W., Schumacher V., von Osten M. (2015). Frontrunners of T cell activation: Initial, localized Ca2+ signals mediated by NAADP and the type 1 ryanodine receptor. Sci. Signal..

[bib32] Woolf E., Grigorova I., Sagiv A., Grabovsky V., Feigelson S.W., Shulman Z., Hartmann T., Sixt M., Cyster J.G., Alon R. (2007). Lymph node chemokines promote sustained T lymphocyte motility without triggering stable integrin adhesiveness in the absence of shear forces. Nat. Immunol..

[bib33] Zanin-Zhorov A., Ding Y., Kumari S., Attur M., Hippen K.L., Brown M., Blazar B.R., Abramson S.B., Lafaille J.J., Dustin M.L. (2010). Protein kinase C-theta mediates negative feedback on regulatory T cell function. Science.

