# Supplementary material for: Durable Interactions of T Cells with T Cell Receptor Stimuli in the Absence of a Stable Immunological Synapse
Source: Cell Rep. 2018 Jan 9;22(2):340–9. doi: 10.1016/j.celrep.2017.12.052 (PMC5775504; doi:10.1016/j.celrep.2017.12.052)
Supplement: Document S1. Supplemental Experimental Procedures, Figures S1–S6, Tables [file mmc1.pdf]

Cell Reports, Volume *22*

Supplemental Information

Durable Interactions of T Cells

with T Cell Receptor Stimuli in the Absence

# of a Stable Immunological Synapse

Viveka Mayya, Edward Judokusumo, Enas Abu Shah, Christopher G. Peel, Willie Neiswanger, David Depoil, David A. Blair, Chris H. Wiggins, Lance C. Kam, and Michael L. Dustin

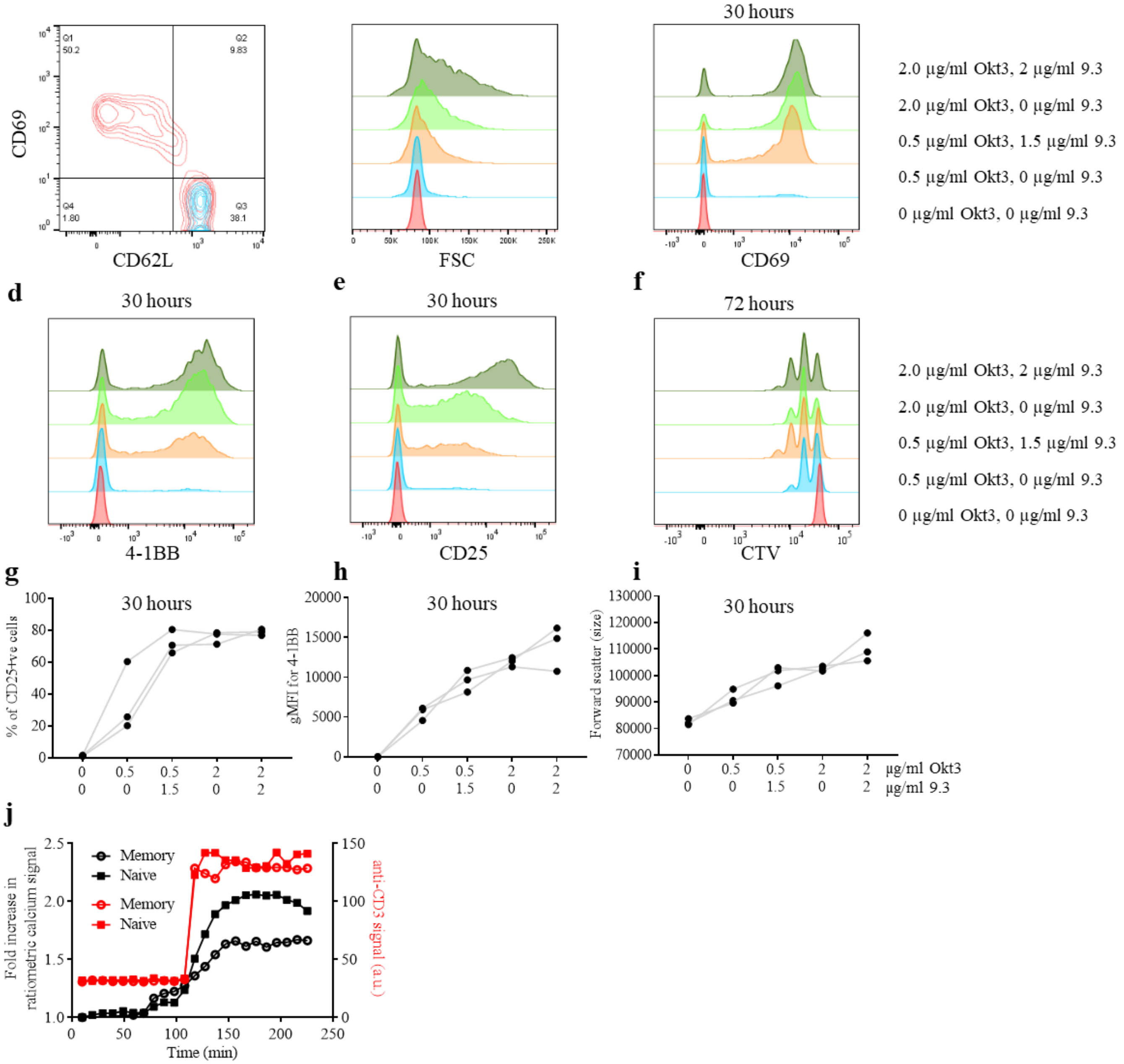

Figure S1: (Related to Figure 3). Priming and activation of human naïve CD8 T cells on stimulatory spots. a-e) Activation status of primed naïve CD8 T cells on stimulatory spots stamped with varying amounts of Okt3 and 9.3 antibodies. The stained surface markers are indicated as well as the duration of interaction with the spots. f) Proliferation of naïve CD8 T cells assayed by dilution of Cell Trace Violet due to cell-division. g to i) Quantification of cytometric information summarised for 3 donors. j) Memory CD8 T cells flux less calcium compared to the naïve cells when they arrest on the stimulatory spots.

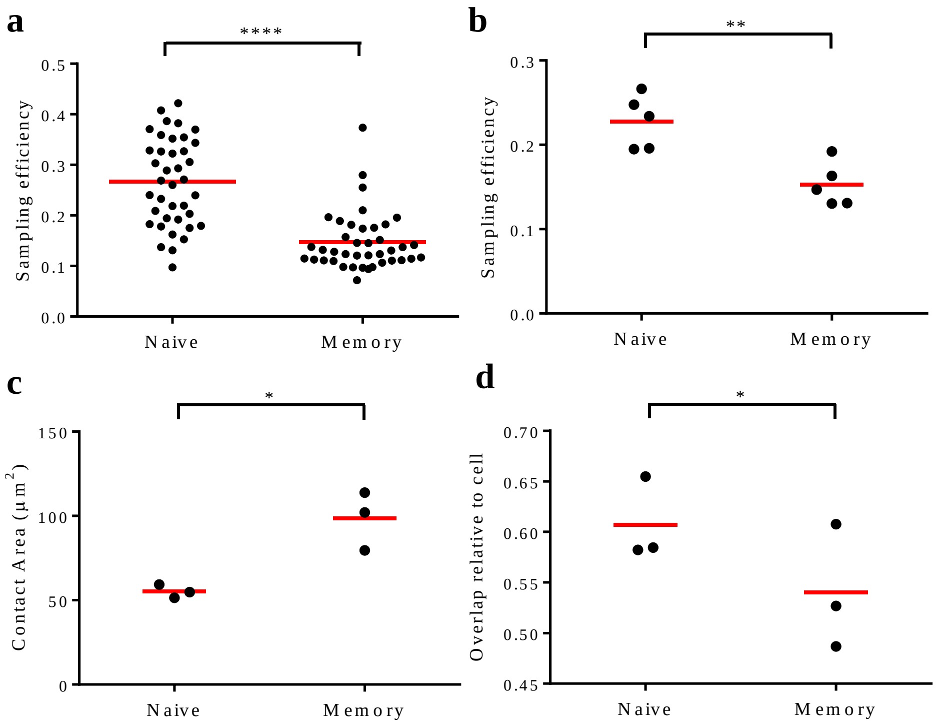

e

Behavior	Study	Name
Motile tendency on DC	Chemokine guidance of central memory cells is critical for anti-viral responses in lymph nodes (PMID: 22980984).	Movie S1
Motile tendency on DC	T cell sensing of antigen dose governs interactive behaviour with Dendritic cells and sets a threshold for T cell activation (PMID: 22980984).	Supplementary video 6, 7, 9
Crawling on DCs	Dynamics of CD8+ T cell priming by Dendritic cells in intact lymph nodes (PMID: 12730692)	Supplementary video 3
Motile tendency on DC	Imaging the single cell dynamics of CD4+ T cells by dendritic cells in lymph nodes (PMID: 15466619)	Video S5, S7

Figure S2: (Related to Figure 4). a and b) Sampling efficiency of naive and memory human CD8 T cells on anti-CD3 and ICAM1 coated glass. Sampling efficiency is defined as the fraction of unique pixels over the total number of pixels underneath the cell-boundary within certain duration of time (20 time-steps or 10 minutes in this case, as in Figure 4b and 4c). A cell with increased motile tendency should have higher sampling efficiency. Data-points in panel a represent individual tracks of cells from a particular donor and in panel b they represent population means from separate blood donors. Statistical significance of difference in population behavior was calculated by Mann-Whitney U-test (in a). Statistical significance of difference in mean values was calculated by paired t-test (in b). c and d) Memory human CD8 T cells have

larger contact area but lesser fractional overlap with the 10 µm stimulatory spots. Mean values from a population of cells from each of the three donors is shown. Statistical significance of difference in mean values was calculated by paired t-test. * for $p \leq 0.05$, ** for $p < 0.01$ and **** for $p < 0.0001$. e) Source of instances in the literature wherein motile tendency of naïve T cells conjugated to antigen-bearing DCs is visible. This has also been noticed by others (Celli et al., 2008).

Description of the videos

Video 1: Behaviour of human CD8 T cells on uniform stimulatory surfaces (Related to Figure 1). Time-lapse video of the motility behaviour of CMRA-labelled naïve (in red) and CMFDA-labelled memory (in green) human CD8 T cells on immobilized OKT3 and ICAM1. Field of imaging was 131.84 µm in size. Progression of time is also noted in the video.

Video 2: Behaviour of human CD8 T cells on cell-sized stimulatory spots (Related to Figure 3). Side-by-side comparison of durability of interaction of naïve (on left) and memory (on right) human CD8 T cells with 10µm stimulatory spots. Spots are shown in magenta. In the upper half, a positive mask of the dilated spot and contact foot-print was applied to the DIC images so that only the cells engaged on the spots were tracked by TIAM. Masked DIC images used for the video here helps with avoiding distraction from motile cells that pass by the spots. In the lower half, the same dataset is shown without the mask. There was some advection in the channel containing naïve cells, as a results of which motile cells have a bias in their movement towards the top of the field. Nonetheless, scanning motility of T cells can be appreciated in the lower half of the video. It is to be noted that advection does not impact durability of interaction. Tracked positions are overlaid as small yellow squares on the masked DIC images. A bigger yellow square flashes to indicate the termination of the track, in other words, signifies dissolution of the synapse and exit from the spot. Memory cells exhibit more such events, implying reduced half-life of interaction on antigenic spots. There were some technical challenges in calculating the half-life of interaction for memory cells, which are detailed in the Image Analysis sub-section of Supplementary Methods. The memory cells that leave a spot, typically engage with another neighboring spot. This can be appreciated by the sizeable number of attached memory cells on spots that were not tracked, as their tracks did not begin at the initially considered frame.

Video 3: Kinapse behaviour highlighted for a single naïve CD8 T cell on a 10µm spot (Related to Figure 4).Time-lapse video of a single naïve CD8 T cell showing prolonged engagement (3 hours) with a 10µm OKT3 spot despite constant generation of protrusions away from the spot. Occasionally, the cell also forms a nascent uropod and a dominant, single protrusion at the opposite end, both of which are transient. The cell is shown as an overlay of DIC and IRM to provide a darker contrast and thus distinguish the concerned cell from other passing-by cells that transiently engage the spot without a juxtaposed contact. Outline of the spot and the cell boundary are also provided on the right side to aid visualization.

Video 4: Kinapse behaviour highlighted for a single naïve CD8 T cell on a 20µm spot (Related to Figure 4).Time-lapse video of a single naïve CD8 T cell showing prolonged engagement (>2 hours) with a 20µm OKT3 spot despite constant kinapse motility along the circumference of the spot. As in video 3, the cell is shown as an overlay of DIC and IRM to provide a darker contrast and thus distinguish the concerned cell from other passing-by cells that transiently engage the spot without a juxtaposed contact. Outline of the spot and the cell boundary are also provided on the right side to aid visualization. The motility of the T cell is seemingly confined or dictated by the boundary of the spot.

Video 5 and 6: Naïve CD8 T cells in prolonged engagement with DCs while exhibiting kinapse behaviour (Related to Figure 4). 3D **t**ime-lapse video of human naïve CD8 T cells (in green) engaging in prolonged interaction with mature monocyte derived DCs (in magenta) that were loaded with Okt3 via their Fc Receptors and embedded in collagen matrix. Motile tendency and protrusive behaviour persists throughout the 1.5 hours of engagement that was captured. We did not observe naïve cells disengaging from the DCs within the 1.5 hours of observation. The migratory movement of other cells in the field was primarily due to CCL19 that was added to the collagen matrix. In video 5, the engaged T cell shifts from one DC to another DC that comes into the vicinity. This implies kinapse mode of interaction with the DCs. In video 6 multiple naïve cells can be seen jostling with motile tendency, yet they stay engaged with the DC. Observations are representative of two separate experiments.

Detailed experimental procedures

Isolation of human T cells

Resting T cells were isolated from leukapheresis products (non-clinical) obtained either from the New York Blood Center (for experiments conducted at the Skirball Institute, NYU Medical Center, New York, USA) or the National Health Service Blood and Transplant Center at the John Radcliffe Hospital (for experiments conducted at the Kennedy Institute of Rheumatology, University of Oxford, Oxford, UK). Total CD8 or CD4 population was enriched using the Rosette Sep (Stemcell Technologies) approach. Naïve and memory cells were isolated by negative selection using the respective EasySep Enrichment Kits (Stemcell Technologies). In some instances memory CD4 cells were isolated by positive selection using CD45RO

microbeads (Miltenyi Biotec) with the flow-through from the LD column used as the naïve CD4 population. Cytometric assessment using CD62L (clone DREG-56, from BD Biosciences) and CD45RO (clone UCHL1, from BD) as surface markers indicated that naïve and memory cells were typically >90% in purity. Cells were cultured for a maximum of 5 days at a density of 3 million/ml in phenol-red free RPMI medium supplemented with 25 mM HEPES, 2 mM glutamine, 1 mM sodium pyruvate, and 10% fetal bovine serum (also used as imaging medium) until imaging.

Isolation of murine CD8 T cells

Cells were obtained from 8-14 week old C57BL/6 (B6) mice or their congenic CD45.1 (commonly referred as SJL mice) counterparts housed in specific pathogen-free conditions. These mice were obtained either from Jackson Laboratory or the NCI mouse repository. OT-I TCR transgenic mice deficient in recombinant-activating gene 1 (RAG1) were bred in-house. All procedures and experiments involving mice were conducted at the Skirball Institute and were approved by the Institutional Animal Care and Use Committee. 1×10^5 OT-I naïve T cells were adoptively transferred into SJL mice by tail vein injection. These SJL mice were infected with 5×10^3 colony-forming units of *Listeria monocytogenes* expressing ovalbumin (Lm-ova) by tail vein injection. The infected mice were used as a source of memory OT-I T cells 30-40 days later. Naïve OT-I T cells were obtained from naïve SJL recipient mice, 1-2 days after transfer of 1×10^6 cells, instead of being directly used. This was done to subject both naïve and memory OT-I T cells to the same procedure of isolation and handling. Polyclonal memory CD8 T cells (defined as $CD44^{hi}$) were obtained from B6 mice that were infected with Lm-ova 30-40 days prior to use. CD44-ve CD8 T cells from the same mice were used as polyclonal naïve CD8 T cells. On the

day of the isolation, the CD8 T cells were first enriched from splenocytes by negative selection using the Dynabeads Untouched Mouse CD8 Cells Kit (Life Technologies/Thermo Fisher). The desired cell populations were then isolated by sorting into cold fetal bovine serum using FACSAria (BD Biosciences). OT1-I T cells were selected as $CD4^-$, $B220^-$, $NK1.1^-$, $MHC\text{-}II^-$, $CD8^+$ and $CD45.2^+$ cells. Polyclonal CD8 T cells were selected as $CD4^-$, $B220^-$, $NK1.1^-$, $MHC\text{-}II^-$ cells with the status of CD44 staining defining the memory and naïve populations as mentioned above. The cells were kept on ice for a maximum of six hours and warmed to 37 °C in imaging medium (see earlier section) for 20 minutes, just before introducing them into the imaging chamber.

The antibodies used for sorting the naïve and memory cells are as follows: CD4 (clone RM4-4, Biolegend), B220 (clone RA3-6B2, Biolegend), NK1.1 (clone PK136, eBioscience), MHC-II (clone M5/114.15.2, eBioscience), CD45.1 (clone A20, Biolegend), CD45.2 (clone 104, eBioscience), CD8α (clone 53-6.7, eBioscience), CD44 (clone IM7, Biolegend).

Preparation of stimulatory surfaces

Uniform coated chambers: Uniform coated surfaces present spatially unlimited ligands adsorbed and immobilized from solution-phase. #1 or #1.5 Labtek 8-well chambers (Nunc) with cover-glass bottom were coated first with 3 µg/ml of recombinant CCL21 (from R&D systems or Peprotech) in 200 µl of phosphate-buffered saline (PBS, pH 7.2) for one hour and then with a solution containing both ICAM1 (ectodomain of murine ICAM1 produced in S2 insect cells and used at 2µg/ml) and anti-CD3 (Okt3 for human cells or 145-2C11 for murine cells, from Ebioscience or BioXcell) in PBS for three hours at 37 °C. In some experiments coating with

CCL21 was omitted. Okt3 was used at 2 µg/ml for a complete response, i.e. for nearly all human naïve CD8 T cells to attach or at 0.05 µg/ml for threshold density of anti-CD3, below which negligible number of human naïve CD8 T cells attach. Anti-CD28 (clone 9.3, BioXcell) and anti-CD8 (Okt8, eBioscience) were used at 1 µg/ml along with ICAM1 and Okt3.

Micro-contact printed chambers: Micro-patterned surfaces presenting spatially limited features of activating and adhesive ligands were prepared by micro-contact printing (Shen et al., 2008a; Shen et al., 2008b). Briefly, topological masters were developed on silicon wafers by patterning a spin-coated layer of poly-methyl methacrylate (PMMA) using electron beam lithography. The following two patterns of circles were defined: 1) 10 µm in diameter, spaced 30 µm center-to-center on a square grid, and; 2) 20 µm in diameter, spaced 50 µm. These patterns were repeated to cover the entire length of the channel of the sticky-Slide VI$^{0.4}$ (Ibidi). The master was then silanized for multiple casting of polydimethylsiloxane (PDMS) elastomer stamps. Sylgard 184 (Dow Corning) PDMS was used for these stamps, mixing 1 part curing agent for 7 parts of the elastomer by mass. Rectangular stamps of PDMS were coated with dye-labeled (using N-Hydroxysuccinimide chemistry) activating antibodies to CD3 (2 µg/ml of Okt3 or 5 µg/ml of 145-2C11) in 150 µl of PBS for one hour. The blocks were then rinsed extensively in PBS, PBS with 0.05% Tween-20 and finally in MilliQ-grade water followed by gentle drying with N2 to remove droplets of water. Borosilicate coverslips (either 24 mm × 40 mm Fisherbrand Cover Glass from Fisher Scientific or 25 mm ×75 mm D 263 M Schott Glass from Ibidi) were pre-cleaned by rinsing sequentially in MilliQ-grade water (10 minutes), 70% ethanol (1 minute) and running deionized water and then baked at 400 °C for 10 hours prior to use as a substrate for micro-contact printing. Anti-CD3 coated PDMS blocks were stamped onto the coverslips for 5

minutes under ~20 g of load for consistency. The patterned coverslip was then affixed to the sticky-Slide VI$^{0.4}$ (Ibidi) and washed sequentially with MilliQ-grade water and PBS. The channels were then coated with 13.5 µg/ml of CCL21 in 30 µl for one hour and then 3 µg/ml of ICAM1 in 180 µl for three hours. The quality and consistency of stamping were confirmed in every case by confocal microscopy, taking advantage of conjugated Alexa Fluor 647 (Molecular Probes) and periodically by macroscopic infra-red imaging (Odyssey Imaging System) of the entire chamber, taking advantage of conjugated IRDye 680LT (LI-COR Biosciences).

Supported Lipid Bilayer chambers: Supported Lipid Bilayers (SLBs) present spatially unlimited and laterally mobile ligands in native state of orientation and structure. Assembly of SLBs presenting UCHT1 Fab' and ICAM1 was conducted essentially as described before, with minor modifications (Choudhuri et al., 2014; Dustin et al., 2007). Small unilamellar liposomes were prepared by extrusion (from Avestin; 100 nm pore size filter) of reconstituted phospholipids (from Aventi Polar Lipids). The molar % of lipids in the liposomes was as follows: 97.5% DOPC, 2.5% DOGS-NTA, and 0.004% DOPE-cap-biotin. The molar fraction of DOPE-cap-biotin was titrated to provide ~30 molecules/µm^2 of UCHT1 Fab'. Bilayers were formed by filling the channels created in the sticky-Slide VI$^{0.4}$ (Ibidi) using 30 µl of liposomes after affixing borosilicate cover-glass (SCHOTT NEXTERION) cleaned with peroxidated H_2SO_4 (piranha solution). ICAM1 was presented at a surface density of ~200 molecules/µm^2.

Cytometry to profile the activation status of naïve T cells

Equivalent numbers of cells as the number of stimulatory spots (~90,000) were introduced into the channel in 30 µl of culture medium. The wells feeding the channel were simultaneously filled

with additional medium using a multi-channel pipette. This prevents displacement of cells from the channel. At various time-points, the all the cells in the channel were collected using ice-cold PBS containing 0.5% BSA and 2mM EDTA, which was found to be effective in dislodging attached cells. These cells were appropriately assayed by flow cytometry for activation markers and cell division by dilution of Cell Trace Violet (CTV). Cells were labelled with 0.3 µM of CTV a priori for measurement of proliferation. The following antibodies were used against the activation markers: CD69 (clone FN50, Biolegend), CD62L (DREG-56, Biolegend), 4-1BB (4B4-1, Biolegend), and CD25 (MA25-1, Biolegend).

Imaging

Imaging in the Labtek 8-well chambers was done with 1 million cells/ml (200 µl) and imaging in the Ibidi sticky-Slide VI$^{0.4}$ channels was done with 3-4 million cells/ml (150 µl) for 10 µm spots and either 5-6 million cells/ml (for crowding on the spots) or 1-2 million cells/ml for 20 µm spots. The cells were pre-treated with inhibitors of PKCθ/α for 20 minutes before using them in the specified experiments in the presence of the inhibitors. Myristoylated pseudosubstrate peptides of PKCα and PKCθ (20 µM; from Calbiochem) inhibit the respective kinases by binding to the active site in a competitive manner. C20 (1 µM) is a compound from Boehringer Ingelheim that acts on PKCθ by non-competitive binding to the active site.

Naïve and memory T cells were imaged together in the same chamber in certain experiments. The cells were differentially labelled with CellTracker dyes CMFDA (at 50 or 100 nM) and CMRA (at 200 or 250 nM) for these experiments. The labelling was in PBS at room temperature for 15 minutes. Cells were washed in PBS before and after the dye labelling. The labelled cells

were used after 2-3 hours and within two days for the experiments. The results were consistent with those obtained without labelling or when the dyes were swapped between the cell types. Calcium imaging was performed by the ratiometric method using the Fluo4-AM (at 3 μM) and Fura Red-AM (at 6 μM) dyes (Wolf et al., 2015). Washed cells were incubated with the dyes for 30 minutes in serum free RPMI medium at 37 °C, washed and further rested for 30 minutes in the imaging medium at 37 °C. Dye-loaded cells were used for imaging within 3 hours.

Cells were imaged using either a Zeiss LSM 510 or an Olympus FluoView FV1200 confocal microscope that was enclosed in an environment chamber (at 37 °C) and operating under standard settings. 40x Plan Neofluar oil immersion objective (1.3 NA) was used on the Zeiss LSM510 and 30x Super Apochromat silicone oil immersion objective (1.05 NA) was used on the Olympus Fluoview FV1200 microscope. Both these lenses are compatible with Differential Interference Contrast (DIC) microscopy. Both microscopes were equipped with a DIC prism to collect DIC images via the transmitted light. Reflected light from the incident laser (typically 543 nm on LSM510 and 635 nm on FV1200) is captured for interference reflection microscopy (IRM). This is facilitated by the flexibility in the choice of dichroics available on these microscopes. Reflected light is captured to record interference that occurs between light reflected from the cover-slip and the closely apposing cell membrane. This provides information on adhesion, spreading, and protrusions of cells. The same laser is used for both IRM and DIC with pixel dwell time of 4 or 8 μsec to minimize photo-toxicity and the build-up of free radicals over hours of acquisition. 8- or 12-bit images of 512-by-512 pixel size were collected at 1x zoom, with an interval of 30 seconds in between. The positions of the micro-contact printed stimulatory spots are recorded just prior to the commencement of the live imaging and not as part of time-

lapse acquisition. Fluorescence channels were included for time-lapse acquisition when the cells were labelled with CellTracker dyes or calcium dyes.

Image analysis

Image analysis and quantification was conducted using TIAM (Tool for Integrative Analysis of Motility), a MATLAB based toolset that we have developed (Mayya et al., 2015). Cells are detected and tracked using the transmitted light images (DIC here). Cell positions are then used to perform local segmentation to features of cells from every image channel. The features include the segmented image itself, outline of the cell in a particular channel and the extracted information such as morphological polarity, contact area and mean fluorescence intensity of the cell. These features and motility-related parameters are stored for every cell position of a track in MATLAB .mat files. We have previously assessed the accuracy of tracking and extracting information using TIAM from similar time-lapse datasets (Mayya et al., 2015). The graphic-user interface of TIAM allows for ensuring that the chosen detection parameters result in good detection. Similarly, individual tracks can be assessed in video-mode. Further, outlines of cells in different channels can be stored and overlaid on the actual images in ImageJ. These visual aids were used when deemed necessary.

IRM+ve parts of tracks were selected prior to the calculation of arrest coefficient. A threshold speed of 0.5 µm/min was used for calculating the arrest coefficient. This threshold was chosen based on the observation that most of the fully sessile human memory CD8 T cells (i.e. with <10 µm net displacement after 2 hours) had an average speed of < 0.5 µm/min. It is to be noted that the speed referred here is not the raw instantaneous speed, but rather a ‘smoothed’ instantaneous

speed by considering displacement over 5 time-steps centered on the time-point of interest. The smoothed speed was considered to reduce the effects of 'wiggle' in the positioning of the centroid that is influenced by protrusive dynamics of the cells.

Pre-processing of images in ImageJ: Microscopy data files are opened as hyperstacks in ImageJ and then converted to 8-bit depth, if necessary. 'Remove Outliers' routine in ImageJ was used to remove constructive interference patterns in IRM images and replace them with pixel values from the neighbourhood. Then the 'Enhance contrast' routine was used to normalize (min-max) the intensity distribution. IRM is very sensitive to focus and planarity drifts, which can lead to spatiotemporal changes in background and foreground intensity. While local segmentation by TIAM is robust to most of these changes, abrupt spatial gradients in background can lead to faulty segmentation. If abrupt spatial gradients were observed, 'bandpass FFT filter' operation was conducted to minimize gradients. The hyperstacks are stored as tiff image series in separate folder for analysis in TIAM.

A positive mask of the dilated spot and contact foot-print was applied to the DIC images so that only the cells engaged on the spots were tracked by TIAM for half-life measurements. Global segmentation was necessary for creating the positive masks. Mask of the spots was created by segmentation using the 'Default' thresholding algorithm in ImageJ. IRM images were processed as described above and the 'Default' thresholding algorithm was used for creating mask of contact foot-prints. Both the masks were dilated by multiple pixels before creating a combined mask by the 'AND' operation between the masks. Such a mask was also applied to the DIC images to quantify the arrest coefficient of cells engaged on 20 µm spots.

A positive mask of the cells was created using the 'Default' thresholding algorirthm in ImageJ on median-filtered image of cells in the Fura-red channel. The positive mask was applied on the ratio image (Fluo-4 over Fura-Red) in 32-bit. It was then converted to 8-bit without loss of information. The ratio image was considered as a fluorescence image by TIAM.

Determination of confinement and positional stability from tracks of cells:

Confinement score is same as probability level L defined previously for the analysis of single particle tracks of membrane proteins (Simson et al., 1995). L was mathematically defined as

$$L = \begin{cases} -\log(\psi) - 1, & when\ \psi \leq 0.1 \\ 0, & when\ \psi > 0.1 \end{cases}$$

Wherein,

$$log\,\psi = 0.2048 - 2.5117Dt/R^2$$

ψ represents the probability that a particle with Brownian diffusion coefficient of D can stay within a distance of R within time t. L was calculated for short track-segments of varying lengths (4 to 10 frames, which defines t) in a sliding window fashion. D was set at 2 μm^2/frame (same as 4 μm^2/min). Largest displacement within the track-segment defines R. Finally, for every position in the track, L is averaged over all track-segments containing that position. Thus, periods with high L represent periods of relative confinement. It is to be noted that value of D changes the inferred values of L, hence the confinement being relative to D. L>3 represents a probability of <0.017 that the relative confinement is by random chance.

Once periods of relative confinement are defined, the algorithm calculates the positional spread within each period. Positional spread is defined as R^2/t, where t is the period of relative

confinement in number of frames and R is the diameter of the confined zone in µm. Diameter of confined zone was calculated based on the average of top-10 most distant pairings of points in the confined zone. A value of <0.666 for R^2/t was found to represent positional stability and all points within the period of relative confinement were assigned the stable state, i.e. value of 1, with default representing kinapse state (value of 0). If the positional spread was above this threshold, then the period of relative confinement was trimmed from the ends until the narrowed period of positional stability was found. If the period of relative confinement was less than 10 frames (5 minutes), it was not considered for positional stability.

Assignment of stable state was found to be largely robust to varying the maximum length of the track segment up to 15 (from 10) and considering a threshold value of 4 for L (from 3). Similarly higher values of D also provided essentially the same results.

Temporal alignment of tracks for the analysis of calcium flux when arresting on the stimulatory spots: Fluorescence intensity of the spots was reported by TIAM only when the local segmentation by TIAM was successful and when the centroid of the cell and the centroid of the segmented area of the spot are less than 1.5 µm apart. This gives an abrupt increase in the spot intensity in the track trace, making manual temporal alignment of multiple tracks feasible. Temporal alignment captures the average behaviour from many cells as they decelerate, arrest and spread onto the stimulatory spot.

Calculation of half-life of interaction on stimulatory spots: Cells that are attached on the spots are the only cells that are present in the finalized images as a result of masking, and

thus are the only cells that are tracked. During the tracking by TIAM, a track gets terminated if the cell leaves the spot, in other words, disappears from the masked image. ‘Survival’ plot of the tracks then provides off-rate and half-life measurements based on approximation of first-order kinetics.

In the benchmark dataset shown in Video 2, manual assessment of exit events revealed that 1 out of the 6 events called by TIAM were wrong for human naïve CD8 T cells and 8 out of 25 events called by TIAM were wrong in the case of human memory CD8 T cells. While the error rate is higher in the case of memory cells, the conclusions are not changed by the higher error. Several factors contribute to higher error in the case of memory cells, which cannot fully be addressed due to trade-off effects in the choice of parameters for detection and tracking: 1) Increased spreading of memory cells necessitates a larger (dilated) area of mask around the spots. However, a larger area also leads to inclusion, detection and tracking of motile cells that are transiently passing by the spots. This can lead to termination of an attached track due to a ‘track-switching’ error. 2) Because of less durable interactions memory cells leave the spot and ‘search’ for another spot to occupy. Thus, there are more instances of memory cells competing among themselves, jostling for space and occupancy on the spot. This also increases the chance of track-switching errors. 3) Increased instances of two or more cells on the same spot necessitate keeping the minimum separation distance allowed between cells to be as low as possible. However, this also increases multi-detection of a single cell.

Quantification of motile tendencies on 10 µm-wide stimulatory spots: Bespoke scripts and functions were written in MATLAB for the calculation of sampling efficiency and

protrusion index. For sampling efficiency of a cell, every pixel in the imaging field that is within the DIC boundary of the cell is marked as visited. For protrusion index, calculation of overlap with the stimulatory spot is the most important aspect, which is done by the AND operation between the segmented image of a cell (in DIC) and that of the stimulatory spot on which it resides. Segmentation of the DIC images of cells and spots was performed using in-built functions in TIAM. Only the arrested cells that do not have any 'neighbor', i.e. spots with a single arrested cell, are considered for the quantification of motile tendencies. This was done to avoid the technical ambiguity in delineating the boundaries of snugged cells and to avoid confounding of results due to influence of one arrested cell on the other.

All the reported statistical analyses were performed with Prism (Graphpad software).

Imaging of T-DC conjugates in collagen gels

Preparation of Monocyte derived DCs: Monocytes were isolated using RosetteSep Monocyte enrichment kit (StemCell #15068) as per the manufacturer's protocol. The monocytes were differentiated into DCs with 50µg/ml GM-CSF and 100µg/ml rhIL-4 (both from Peprotech) for 4 days in complete RPMI, then the following mixture of inflammatory cytokines was added for another 24hrs to induce maturation: 40ng/ml TNFα, 20ng/ml IFNγ, 10ng/ml IL1β (all from Peprotech) and 1µg/ml PGE2 (from Sigma).

Cell labelling: T-cells were labelled with 250 nM DeepRed Cell tracker (Invitrogen # C34565) for 20min at 37C in complete RPMI. The DCs were labelled similarly with 250 nM CMFDA (Invitrogen # C7025). The cells were used immediately for downstream experiments.

Loading of DCs with anti-CD3: Mature DCs were collected on day 5 after isolation and their concentration readjusted to 10^6/ml. Anti-CD3 (clone Okt3; 1 µg/ml) was added to the DCs and left to be capture by their Fc receptors for 30min at 37C. The DCs were then washed and used for stimulating T-cells.

3D collagen culture: Collagen master mix was prepared by mixing 75 µl of 3 mg/ml bovine collagen I (Cell Systems), with 10 µl 10xMEM and 5 µl of 7.5% sodium bicarbonate and CCL19 to a final concentration of (1 µg/ml). 200,000 T-cells and an equivalent number of DCs were mixed with the collagen master mix to reach a final concentration of 1.7mg/ml collagen. 50 µl of this collagen and cell mixture is introduced into a channel of VI Ibidi chamber and left to polymerise for 30-60mins at 37 ºC in an inverted position to prevent the cells from settling down on the coverslip. The wells feeding the channel were toped up with complete media and the sample is taken for 4D imaging.

Imaging: The collagen gel cell cultures were mounted on a PerkinElmer spinning disk fitted with CSU-10 head and a flash 4.0 sCMOS camera and enclosed in an environmental chamber (37 ºC and 5% CO2). Imaging was done using a 30x silicone oil objective from Olympus. Z-stacks were taken at 3 µm intervals and the time-lapse is recorded at 1 min^{-1}.

References

Celli, S., Garcia, Z., Beuneu, H., and Bousso, P. (2008). Decoding the dynamics of T cell-dendritic cell interactions in vivo. Immunological reviews *221*, 182-187.
Choudhuri, K., Llodra, J., Roth, E.W., Tsai, J., Gordo, S., Wucherpfennig, K.W., Kam, L.C., Stokes, D.L., and Dustin, M.L. (2014). Polarized release of T-cell-receptor-enriched microvesicles at the immunological synapse. Nature *507*, 118-123.
Dustin, M.L., Starr, T., Varma, R., and Thomas, V.K. (2007). Supported planar bilayers for study of the immunological synapse. Current protocols in immunology *Chapter 18*, Unit 18.13.
Mayya, V., Neiswanger, W., Medina, R., Wiggins, C.H., and Dustin, M.L. (2015). Integrative analysis of T cell motility from multi-channel microscopy data using TIAM. Journal of immunological methods *416*, 84-93.
Shen, K., Qi, J., and Kam, L.C. (2008a). Microcontact printing of proteins for cell biology. Journal of visualized experiments : JoVE.
Shen, K., Thomas, V.K., Dustin, M.L., and Kam, L.C. (2008b). Micropatterning of costimulatory ligands enhances CD4+ T cell function. Proceedings of the National Academy of Sciences of the United States of America *105*, 7791-7796.
Simson, R., Sheets, E.D., and Jacobson, K. (1995). Detection of temporary lateral confinement of membrane proteins using single-particle tracking analysis. Biophysical journal *69*, 989-993.
Wolf, I.M., Diercks, B.P., Gattkowski, E., Czarniak, F., Kempski, J., Werner, R., Schetelig, D., Mittrucker, H.W., Schumacher, V., von Osten, M.*, et al.* (2015). Frontrunners of T cell activation: Initial, localized Ca2+ signals mediated by NAADP and the type 1 ryanodine receptor. Science signaling *8*, ra102.
