# Supplementary material for: Durable Interactions of T Cells with T Cell Receptor Stimuli in the Absence of a Stable Immunological Synapse
Source: Cell Rep. 2018 Jan 9;22(2):340–9. doi: 10.1016/j.celrep.2017.12.052 (PMC5775504; doi:10.1016/j.celrep.2017.12.052)
Supplement: Document S2. Article plus Supplemental Information [file mmc8.pdf]

Cell Reports

Report

Durable Interactions of T Cells with T Cell Receptor Stimuli in the Absence of a Stable Immunological Synapse

Graphical Abstract

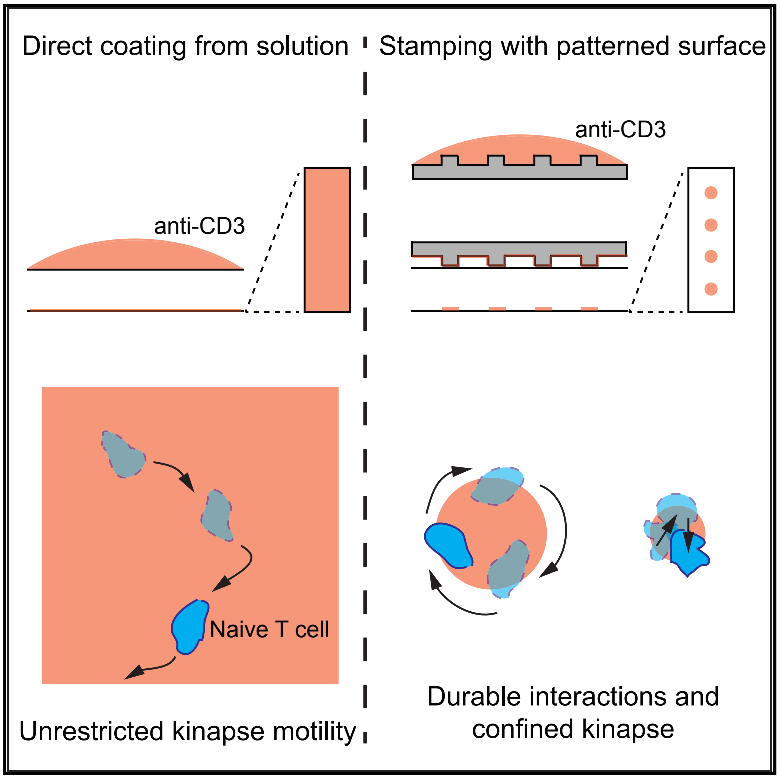

Highlights

- Naive T cells spend more time in the motile kinapse state
- Only human memory CD8 T cells spend more time in the stable synapse state
- Kinapses do not reduce durability of interaction with cell-sized stimulatory spots
- Spatial restriction of TCR stimulation does not force formation of a synapse

Authors

Viveka Mayya, Edward Judokusumo, Enas Abu Shah, ..., Chris H. Wiggins, Lance C. Kam, Michael L. Dustin

Correspondence

michael.dustin@kennedy.ox.ac.uk

In Brief

T cells primarily form two types of adhesive junctions when interacting with stimulatory surfaces: stable synapses and motile kinapses. Mayya et al. demonstrate that durable interactions with antigen do not require formation of a stable synapse.

Mayya et al., 2018, Cell Reports 22, 340–349
January 9, 2018 © 2017 The Author(s).
https://doi.org/10.1016/j.celrep.2017.12.052

OPEN ACCESS
CellPress

Cell Reports
Report

Durable Interactions of T Cells with T Cell Receptor Stimuli in the Absence of a Stable Immunological Synapse

Viveka Mayya,[1,2] Edward Judokusumo,[3] Enas Abu Shah,[1] Christopher G. Peel,[1] Willie Neiswanger,[4,5] David Depoil,[1,6] David A. Blair,[2,7] Chris H. Wiggins,[5] Lance C. Kam,[3] and Michael L. Dustin[1,2,8,*]
[1]Kennedy Institute of Rheumatology, University of Oxford, Oxford OX3 7FY, UK
[2]Skirball Institute of Biomolecular Medicine, New York University School of Medicine, New York, NY 10016, USA
[3]Department of Biological Engineering, Columbia University, New York, NY 10027, USA
[4]Machine Learning Department, Carnegie Mellon University, Pittsburgh, PA 15213, USA
[5]Department of Applied Physics and Applied Mathematics, Columbia University, New York, NY 10027, USA
[6]Present address: Immunocore Limited, Abingdon OX14 4RY, UK
[7]Present address: Boehringer Ingelheim Corporation, Ridgefield, CT 06877, USA
[8]Lead Contact
*Correspondence: michael.dustin@kennedy.ox.ac.uk
https://doi.org/10.1016/j.celrep.2017.12.052

SUMMARY

T cells engage in two modes of interaction with antigen-presenting surfaces: stable synapses and motile kinapses. Although it is surmised that durable interactions of T cells with antigen-presenting cells involve synapses, *in situ* 3D imaging cannot resolve the mode of interaction. We have established *in vitro* 2D platforms and quantitative metrics to determine cell-intrinsic modes of interaction when T cells are faced with spatially continuous or restricted stimulation. All major resting human T cell subsets, except memory CD8 T cells, spend more time in the kinapse mode on continuous stimulatory surfaces. Surprisingly, we did not observe any concordant relationship between the mode and durability of interaction on cell-sized stimulatory spots. Naive CD8 T cells maintain kinapses for more than 3 hr before leaving stimulatory spots, whereas their memory counterparts maintain synapses for only an hour before leaving. Thus, durable interactions do not require stable synapses.

INTRODUCTION

T cell priming requires hours of interaction with cognate antigen-presenting cells (APCs) (Iezzi et al., 1998). Results from *in vivo* imaging suggest that T cells decelerate and arrest on APCs to achieve this duration of signaling when antigen is spatially limited (Mempel et al., 2004; Miller et al., 2004) but can continue to migrate throughout the APC network when antigen is present on many contiguous APCs (Friedman et al., 2010; Hugues et al., 2004; Moreau et al., 2012; Sims et al., 2007). *In vitro* studies indicate two modes of interaction of T cells with APCs that may account for these observations: symmetric and stable synapses and asymmetric and motile kinapses (Dustin, 2007; Friedman et al., 2010; Sims et al., 2007). Decelerated movement of T cells *in vivo* within networks of stimulatory APCs arises from kinapses. Generally, durable interactions of T cells *in vivo* with spatially isolated stimulatory APCs are interpreted to arise from synapses. However, whether durable interactions are mediated by synapses or confined kinapses is not ascertainable because of the inability to resolve details of the interface over time. This inability is mainly a result of internal tissue movement and inherent limitations of 3D rendering. Synapses and kinapses have functional implications, with synapses being more efficient for effector functions (Beal et al., 2008; Huse et al., 2006) and kinapses allowing greater exploration of local networks (Moreau et al., 2015). However, it has been proposed that the polarized distribution of the motility apparatus along the plane of contact in the kinapse mode limits the durability of interaction (Davis, 2009; Dustin, 2007; Gunzer et al., 2000; Moreau and Bousso, 2014). Our goal in this study was to establish an *in vitro* platform under optically ideal settings to determine cell-intrinsic modes of interaction of different T cell subsets when faced with continuous or spatially restricted stimulation and to examine the relationship between the mode and durability of interaction.

We have studied the cell-intrinsic behavior of freshly isolated human and mouse T cells using 2D stimulatory surfaces on glass supports because of the ideal optics. Spatially continuous stimulatory surfaces are based on classical coating approaches or supported planar lipid bilayers (SLBs) presenting ICAM1 and anti-CD3 (Dustin et al., 1997; Parsey and Lewis, 1993). Using such 2D substrates, we found that human naive CD8, human naive and memory CD4, and murine naive and memory CD8 T cells all spend more time in the kinapse mode. Only human memory CD8 T cells formed a majority of synapses. To quantify the duration of interaction with spatially limited stimulation, we combined a 2D chemokinetic substrate composed of ICAM1 and CCL21 (Woolf et al., 2007) with discrete spots of anti-CD3 formed by micro-contact printing (Shen et al., 2008b). This system recapitulates the basic features necessary for T cell scanning, deceleration, and durable interactions observed *in vivo*. Surprisingly, we did not observe the expected inverse

 © 2017 The Author(s).
This is an open access article under the CC BY license (http://creativecommons.org/licenses/by/4.0/).

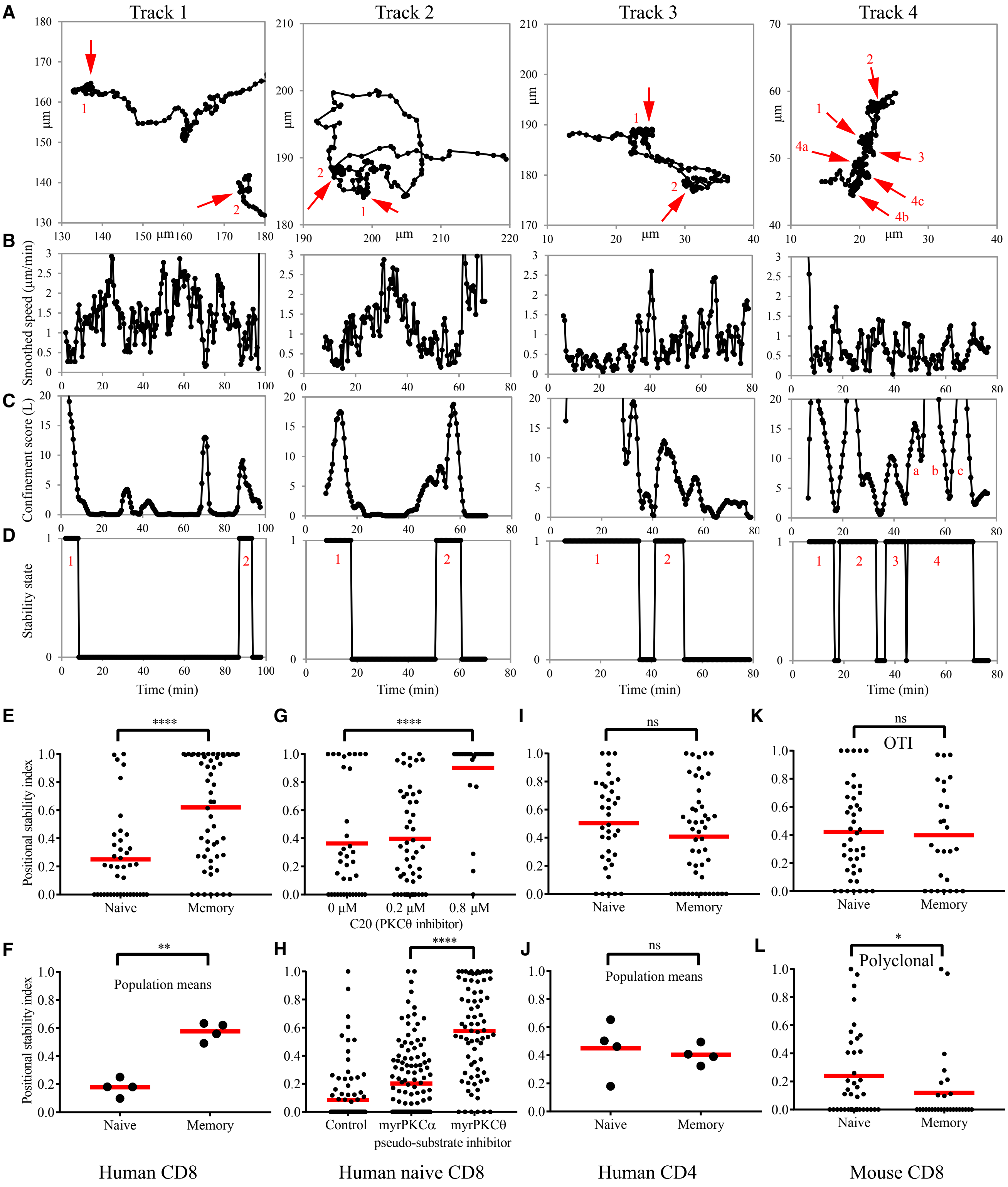

Figure 1. All Major Resting T Cell Subsets, Except Human CD8 Memory Cells, Spend More Time in the Kinapse Mode during Interaction with Uniformly Coated Stimulatory Surfaces

(A) Four representative tracks of human naive CD8 T cells. Periods of positional stability, presumably corresponding to synapse mode of interaction, are highlighted using red arrows and numbered for reference. Additional periods of positional stability that one may visually infer are either of too short a duration or represent drastic turns during motility.

(legend continued on next page)

correlation between kinapses and durability of interaction. Further, we found that kinapse motility is intact on the stimulatory spots even as naive T cells undergo durable interactions over hours and that spatial restriction of anti-CD3 did not force formation of a stable synapse or exit from the anti-CD3 spots. This result demonstrates that naive T cells can achieve durable interactions for priming without forming stable synapses.

RESULTS

Migratory Response of T Cell Subsets to TCR Stimulation

We first utilized an established 2D T cell migration platform to test the cell-intrinsic tendency of T cell subsets to form synapses and kinapses in response to TCR ligation. Although prior studies suggest that human mixed naive and memory CD4 T cells form kinapses in 2D (Zanin-Zhorov et al., 2010) and 3D (Gunzer et al., 2000) settings, a systematic analysis of naive and memory subsets from peripheral blood has not been undertaken. Glass surfaces were uniformly coated with anti-CD3ε and ICAM1, and the migration of freshly isolated T cells was tracked over a period of 2 hr. Initially we used 2 μg/mL of anti-CD3ε for adsorption, which resulted in a surface density that caused nearly all cells to form adhesive contacts (Movie S1). Human naive CD8 T cells were seen to exhibit periods of positional stability that are synonymous with the stable synapse as well as motility that is characteristic of the kinapse mode (Figure 1A; Movie S1; Sims et al., 2007). To identify these periods of positional stability and motility, we first considered the speed of the cells (Figure 1B). Although the speed was generally reduced during periods of positional stability, it was hard to demarcate these periods with any certainty because of the small range of speed within which the cells move and because of constant, abrupt changes in speed. Therefore, we implemented an algorithmic approach to identify the transient, relative confinement that was originally developed for the analysis of single-particle tracks of membrane proteins (Simson et al., 1995; Figure 1C). We then determined the positional spread during periods of relative confinement to confirm whether the cell was in synapse mode (Figure 1D; Supplemental Experimental Procedures). After ascertaining the mode of interaction at every instance, we calculated the "positional stability index," which is defined as the fraction of time the cell spends in the stable synapse state. This parameter was used to quantify the intrinsic tendency of the T cells to form synapses instead of kinapses or vice versa.

Human naive CD8 T cells formed kinapses, whereas the memory counterparts spent more time in the synapse mode, based on analysis of individual cells from a single donor (Figure 1E; Movie S1), and when mean values from 4 donors (Figure 1F) were considered. To relate this to prior observations, we investigated the role of protein kinase Cθ (PKCθ), which promotes kinapse motility in murine naive CD4 T cells *in vitro* and *in vivo* (Sims et al., 2007). Small-molecule (Figure 1G) or peptide-based inhibitors (Figure 1H) of PKCθ increased the positional stability index for the naive CD8 T cells. Thus, the motility observed in this model system is analogous to kinapses observed *in vivo* and on SLBs. We expanded our observations to other human and mouse T cells subsets. Naive and memory human CD4 T cells spent more time in the kinapse mode both within a population of cells (Figure 1I) and between 4 donors (Figure 1J). Furthermore, naive and anti-*Listeria* memory CD8 T cells from OT-I TCR transgenic mice (Figure 1K) or polyclonal *Listeria*-specific memory CD8 T cells from B6 mice (Figure 1L) all formed kinapses. OT-I naive T cells are known to form kinapses on SLBs presenting peptide-major histocompatibility complex (pMHC) and ICAM1 and *in vivo* upon intravenous (i.v.) injection of the cognate peptide (Friedman et al., 2010). Furthermore, the distribution of migratory speed of P14 T cell receptor (TCR) transgenic naive and memory T cells in the presence of antigen-loaded dendritic cells (DCs) is also reflective of kinapse motility *in vivo* for both subsets (Sung et al., 2012). Overall, across all populations, only human memory CD8 T cells exhibited an intrinsic tendency to form synapses for a longer duration, whereas the other human and mouse subsets we examined spent more time in the kinapse mode.

We wanted to further investigate the contrasting tendencies of naive and memory human CD8 T cells under different conditions. We first assessed the mode of interaction on SLBs presenting fluorescently labeled anti-CD3 Fab′ and ICAM1. In this model, kinapse motility can be easily identified through the trail of TCR-enriched micro-vesicles shed by migrating cells, whereas cells with stable synapses maintain TCR micro-vesicles within the interaction interface (Choudhuri et al., 2014). Naive human CD8 T cells spent more time in the kinapse mode, and memory CD8 T cells predominantly formed synapses on SLBs (Figures 2A–2C). This was found to be the case even at the drastically reduced density of anti-CD3 adsorbed to glass (Figures 2D and 2G). Although the homeostatic lymphoid chemokine

(B) The speed of the cells shown in (A) over the duration of the tracks. The speed plotted here is smoothed by averaging the instantaneous speed over the two frames before and after the frame in question. However, the profile shows abrupt changes and fails to demarcate the periods of positional stability.
(C) The confinement score (originally termed probability level, L) identifies the periods of relative confinement (Simson et al., 1995). L > 3 represents a probability of < 0.017 that the confinement is due to random chance, which is the threshold value used to demarcate periods of relative confinement.
(D) The positional spread within each period of relative confinement is considered to determine whether the cell was in the stable state (value of 1) that is synonymous with synapse mode of interaction. Positional spread is defined by R^2/t, where t is the period of relative confinement, and R is the diameter of the confined zone. A value of < 0.666 μm^2/frame was found to represent positional stability. The fraction of time the cell spends in the stable state is called the positional stability index. Thus, a positional stability index of > 0.5 means that the cell has spent more time in stable synapse mode than in motile kinapse mode and vice versa.
(E–L) The positional stability index of resting T cell subsets on coverglass uniformly coated with anti-CD3 and ICAM1. The type of T cell subset examined is denoted at the bottom or in the panels and in the category names of dot plots (for example, naïve and memory cells from human CD8 T cells in E and human CD4 T cells in I). Inhibitors of PKCθ shift the balance from kinapses toward synapses in human naive CD8 T cells (G and H). The data points in (F) and (J) represent population means from separate blood donors, whereas, in rest of the panels, they represent individual tracks of cells from a particular donor. Mean values are given as red horizontal bars. The data shown in (G), (H), (K), and (L) are representative of two independent experiments.

OPEN
ACCESS
CellPress

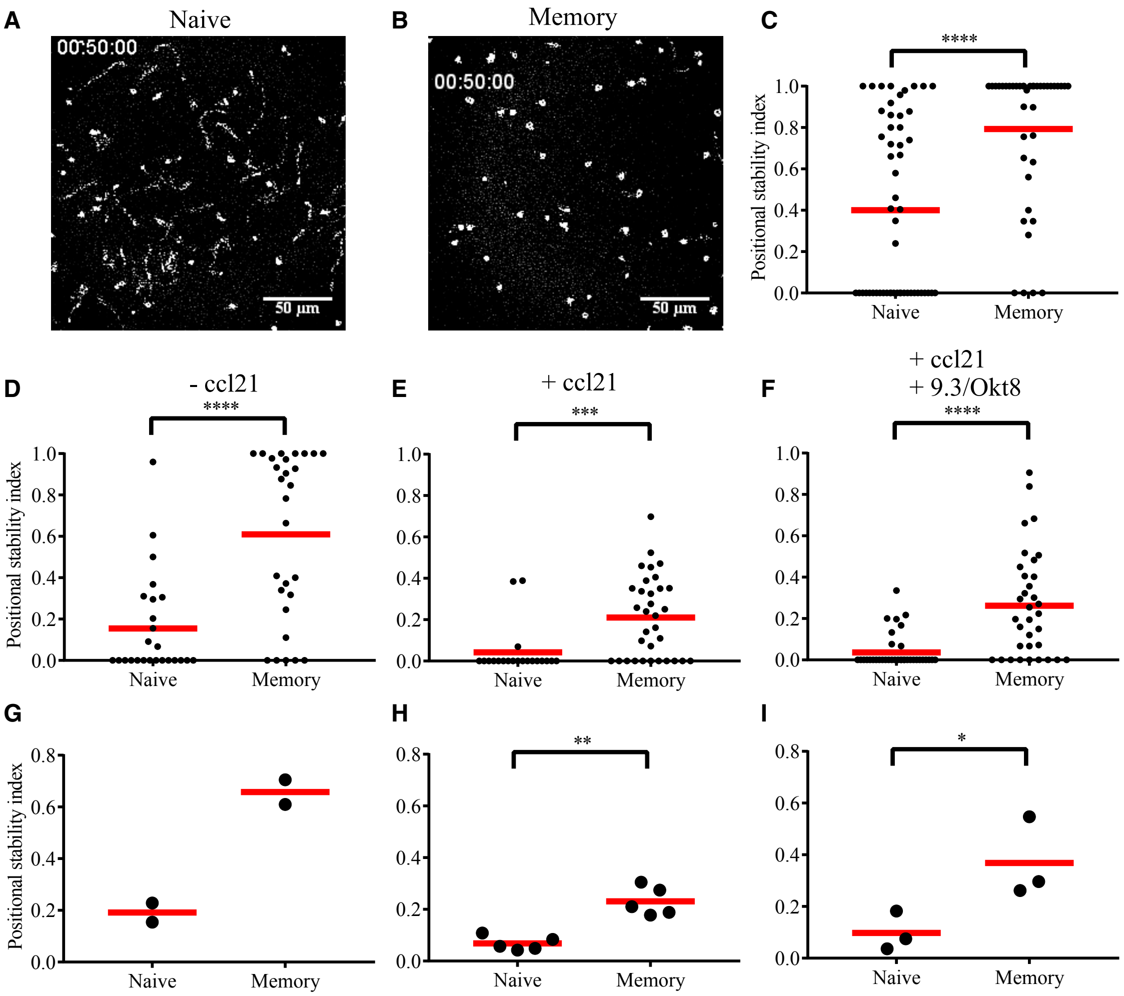

Figure 2. The Lower Positional Stability Index of Human Naive CD8 T Cells Remains Intact Even When the Characteristics of the Stimulating Surface Change

(A and B) Micrographs of fluorescent UCHT1 Fab′ taken 50 min after the cells were introduced on SLBs presenting UCHT1 Fab′ and ICAM1 as freely mobile ligands. The images show TCR clusters confined to the interface (B) because of the synapse of memory cells and trail of UCHT1 (A) shed by kinapses of naive cells. The data shown are representative of two independent experiments.

(C) Positional stability index of cells quantified from tracks on SLBs.

(D–I) Positional stability index of human CD8 T cells on coverglass presenting the threshold density of immobilized OKT3, below which very few naive cells respond by attaching. The presence of additional ligands (CCL21 in E, F, H, and I and 9.3 and OKT8 antibodies in F and I, with no additional ligand in D) is noted at the top. Immobilized ICAM1 was present in all experiments. The data points in (G), (H), and (I) represent population means from separate blood donors, whereas, in the rest of the panels, they represent individual tracks of cells from a particular donor. Mean values of plotted data points are given as red horizontal bars.

CCL21 reduces the time T cells spend in synapse mode, memory CD8 T cells consistently spend more time in synapse mode than naive cells (Figures 2E, 2F, 2H, and 2I). The behavior of naive cells did not change appreciably when costimulatory and co-receptors were also engaged using antibodies (Figures 2F and 2I). Together, we conclude that, with the exception of human memory CD8 T cells, all other resting T cell subsets we examined have a cell-intrinsic tendency to predominantly form kinapses on uniform stimulatory surfaces.

Durability of Interaction on Stimulatory Spots

We then tested whether kinapse-based motility leads to a reduced duration of interaction on spatially limiting and distributed stimulatory spots created by micro-contact printing (Figure 3A). Patterned surfaces have previously been used to explore the influence of different spatial patterns of ligands on the extent of T cell activation (Doh and Irvine, 2006; Shen et al., 2008b). In our case, the micro-patterned antigen-presenting surface was particularly inspired by and designed to emulate the spacing of individual antigen-loaded DCs within the network of DCs in lymph nodes (Lindquist et al., 2004). Micro-patterned printing of anti-CD3 provides stimulatory spots with biophysical and biochemical characteristics very similar to uniform stimulatory surfaces. Thus, it is expected that both initiate the same mode of triggering of TCRs and allow for direct comparison of results from the two model stimulatory surfaces. An additional

OPEN
ACCESS
CellPress

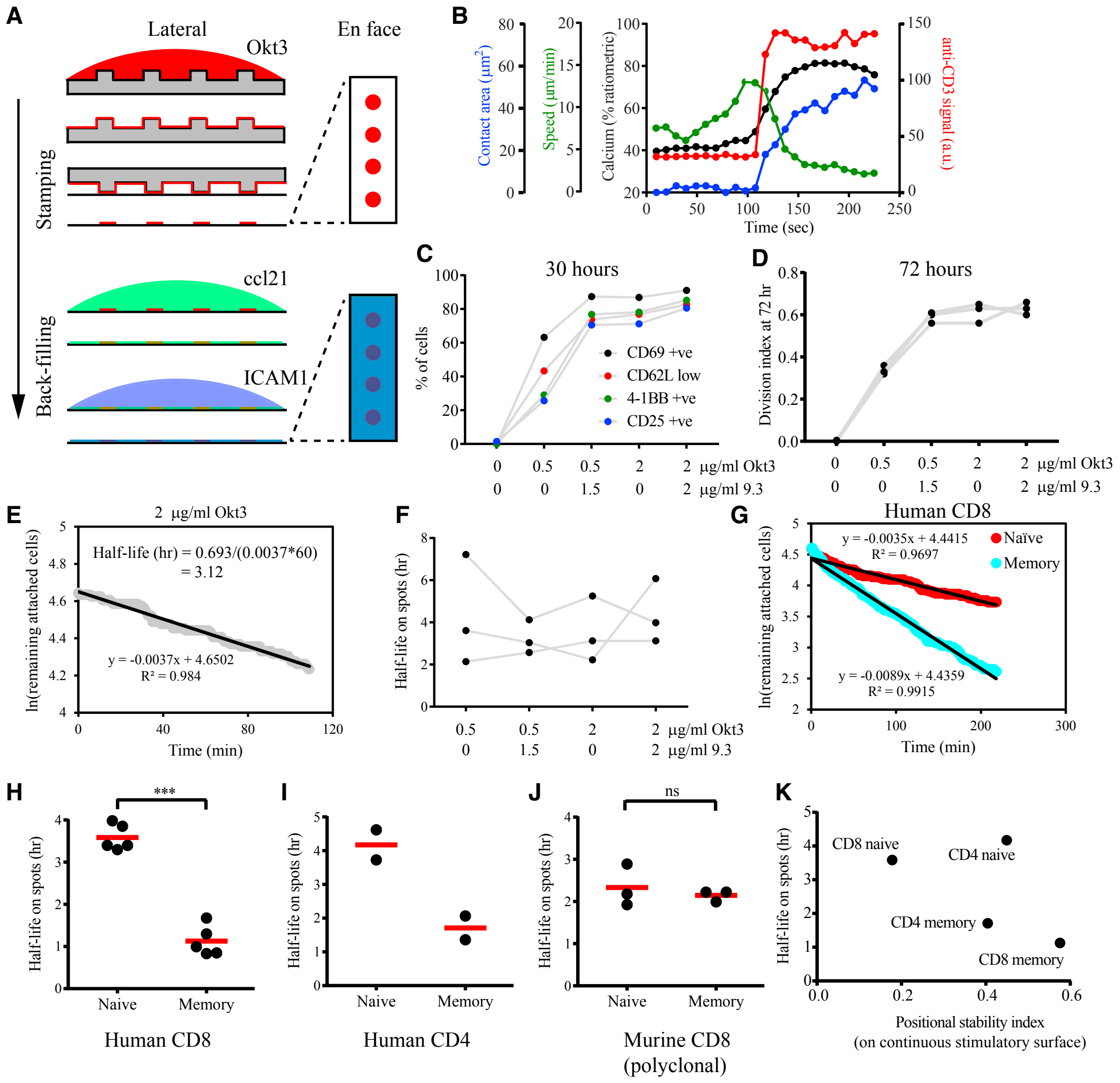

Figure 3. The Durability of Interaction of Human T Cell Subsets with Spatially Limiting Stimulatory Spots Does Not Correlate with the Arrest Coefficient

(A) Schematic of the micro-contact printing procedure for making stimulatory spots that emulate the spatially limiting and distributed nature of antigen presentation in lymph nodes with pervasive adhesion ligands and homeostatic chemokines. Anti-CD3ε is adsorbed on to the polydimethylsiloxane (PDMS) cast with patterned indentations. The PDMS is then stamped to transfer some of the adsorbed protein onto the coverglass. The entire surface is then coated sequentially with the chemokine CCL21 and ICAM1. See Experimental Procedures for details.

(B) Calcium influx (in black), arrest (in green), and spreading (in blue) of motile human naive CD8 T cells on the stimulatory spots (in red) captured by temporally aligning tracks of cells as they reach and arrest on the spots. Average values from 20 cells based on such a virtual synchronization are shown.

(C) Activation status of cells after 30 hr of interaction with the stimulatory spots presenting varying amounts of Okt3 and 9.3 antibodies. See also Figure S1.

(D) Proliferation of naive CD8 T cells from 3 donors after 72 hr of interaction with the stimulatory spots presenting varying amounts of Okt3 and 9.3 antibodies. The division index is the average number of divisions for all cells present.

(E) Illustration for the calculation of the half-life of interaction with stimulatory spots. T cells are introduced to find and arrest onto the spots. Live-cell imaging commences when at least ~50% of the spots are occupied by arrested cells. The cells found to be initially arrested on the spots are tracked through the time lapse, and the percentage of cells remaining on the same spots is tallied. Natural logarithmic transformation of the percentage of remaining cells provides a curve with a very good linear fit. The half-life of interaction is calculated by the formula ln(2)/slope according to first-order kinetics.

(F) Co-stimulation by the 9.3 antibody did not influence the half-life of interaction with stimulatory spots.

(G) A larger percentage of memory CD8 T cells (in cyan) leave the spot onto which they had initially arrested, reflecting a shorter half-life of interaction. It is to be noted that memory cells, or, for that matter, naive cells, that leave a spot typically engage and attach onto another neighboring spot. However, such re-engagements are disregarded in this “survival” analysis.

(H–J) Half-life of interaction of specified T cell subsets upon attachment. The type of T cell subset examined is denoted at the bottom and in the category names of the dot plots (human CD8s in H, human CD4s in I, and murine CD8s in J). Human naive T cells have an appreciably longer half-life of interaction

(legend continued on next page)

critical feature of the T cell area in the lymph node is the presentation of the homeostatic chemokine CCL21 by stromal cells and lymphoid-resident DCs, which prompts the scanning motility of naive T cells (Bousso, 2008). Glass-adsorbed CCL21 has been shown to prompt a similar persistent motility in T cells (Woolf et al., 2007). Adoption of this approach with micro-contact printing allows the T cells to efficiently locate and interact and engage with the stimulatory spots and mimic the main aspects of the *in situ* environment in which naive T cells are primed.

Human naive CD8 T cells scanned the surface, decelerated, arrested, and attached to the 10-μm stimulatory spots placed 30 μm apart on a square grid (Figure 3B; Movie S2, bottom left quadrant). Robust intracellular calcium flux was also observed as the cells arrested and spread, indicating productive suprathreshold TCR signaling. We then asked whether the naive T cells are indeed primed on the stimulatory spots. 50% of the cells were found to have expressed CD69 and shed CD62L after 12 hr (Figure S1A). By 30 hr, 80% of the cells express CD69, along with other activation markers such as CD25 and 4-1BB (Figure 3C; Figure S1G). By 72 hr, the cells had divided at least twice (Figure S1F). Thus, anti-CD3 alone on the stimulatory spots is sufficient to prime naive CD8 T cells. We assessed the influence of co-stimulation by stamping the 9.3 antibody clone against CD28 along with Okt3. A robust effect of co-stimulation was observed only when 0.5 μg/mL of Okt3, but not 2 μg/mL of Okt3, was used for stamping (Figures 3C and 3D; Figures S1B–S1I). Also, increasing the concentration of Okt3 from 0.5 to 2 μg/mL obviated the need for co-stimulation during priming. By following the fate of attached cells regarding when they exit the stimulatory spots, we can calculate the half-life of interaction (Figure 3E). Surprisingly, we did not observe any decisive contribution of co-stimulation toward the durability of interaction with the stimulatory spots (Figure 3F). Therefore, for all ensuing experiments comparing naive and memory T cells, we considered anti-CD3 alone (at 2 μg/mL) on the stimulatory spots.

We found that the human naive CD8 T cells exited the spots at a slower rate than memory CD8 T cells (Figure 3G; Movie S2, top half). Human naive CD8 T cells were found to interact with a half-life of 3.6 hr, whereas the memory CD8 T cells had a half-life of 1.1 hr on 10-μm spots (Figure 3H). Similar results were obtained in the human CD4 subsets (Figure 3I). Both human CD4 and CD8 memory cells have less durable interactions on the stimulatory spots. Both of these subsets are known to have reduced phosphorylation of TCR-proximal signaling proteins and reduced calcium levels (Adachi and Davis, 2011). Accordingly, we observed reduced calcium influx in memory CD8 T cells on the stimulatory spots (Figure S1J). This offers a likely explanation for the less durable interaction of memory cells. Murine polyclonal CD8 naive and anti-*Listeria* memory T cells were found to have an intermediate half-life of ~2 hr on 10-μm spots (Figure 3J). This result is consistent with *in vivo* observations wherein adoptively transferred naive and *ex vivo*-generated memory P14 TCR transgenic CD8 T cells engaging with lipopolysaccharide (LPS)-activated DCs that migrate from the footpad into the popliteal lymph node were found to have the same contact duration with the DCs (Sung et al., 2012). Overall, we did not observe the expected positive correlation between positional stability index on uniform stimulatory surfaces and durability of interaction on stimulatory spots (Figure 3K).

The Motile Tendency of Kinapses Is Intact on Stimulatory Spots

We considered the possibility that the behavior of naive cells is fundamentally different on the spatially confined stimulatory spots, which might force formation of synapses. For this, we focused on comparison of human naive and memory CD8 T cells because they had exhibited a stark dichotomy in behavior (Figures 1 and 3). Visual examination of the dynamics of naive cells engaged on 10-μm-wide stimulatory spots revealed that the motile tendency generated by kinapses is intact and that the cells continuously explore new areas (Movie S3, for example). To quantify this, we defined a "sampling efficiency" parameter that measures the fraction of unique pixels within the cell outline over 20 frames or 10 min (Figure 4A). Naive cells exhibited a significantly greater sampling efficiency than the memory counterparts on the spots (Figures 4B and 4C), as observed on the uniformly coated surfaces (Figures S2A and S2B). We found that naive cells displayed significantly greater sampling over a range of frame numbers and intervals (data not shown). We also noted that the naive cells displayed continuous protrusions in different directions away from the spot; however, they still remained on the spot, apparently because of interspersed preferential retraction from the non-stimulatory area (Movie S3). To quantify this behavior, we defined a "protrusion index" parameter that measures the fractional area of the cell that is outside of the spot, taking into account variations in the projected area of the cell relative to the size of the spot (Figures 4D and 4E). The naive cells exhibited a significantly higher protrusion index than memory cells (Figures 4F and 4G). Thus, quantification of the motile tendencies in human naive and memory CD8 T cells revealed that the intrinsic behavior observed on uniform stimulatory surfaces is preserved on 10-μm stimulatory spots. Dynamic sampling and protrusive behavior were also observed in all the other resting T cell subsets examined, suggesting that they all retained their kinapse tendencies on 10-μm spots (data not shown).

Human memory CD8 T cells form larger contacts on uniformly coated surfaces (Movie S1) and on 10-μm-wide spots (Figures 4E; Figure S2C). The memory cells also have a lesser portion of the cell surface exposed to the stimulatory spots (Figure S2D). Thus, it is possible that memory cells actively decide to dissolve the synapse on account of sub-optimal surface exposure on 10-μm spots, leading to under-estimation of the potential for durable interactions. Therefore, we extended the comparisons to 20-μm stimulatory spots. Many human naive CD8 T cells were seen to circle along the edges on the 20-μm-wide spots because

compared with the memory counterparts. Mean values of plotted data points are given as red horizontal bars. Each half-life measurement shown here came from different donors.

(K) Relationship between the half-life of interaction on stimulatory spots and the positional stability index on a continuous stimulatory surface among human T cell subsets. Mean values from all the donors examined are plotted.

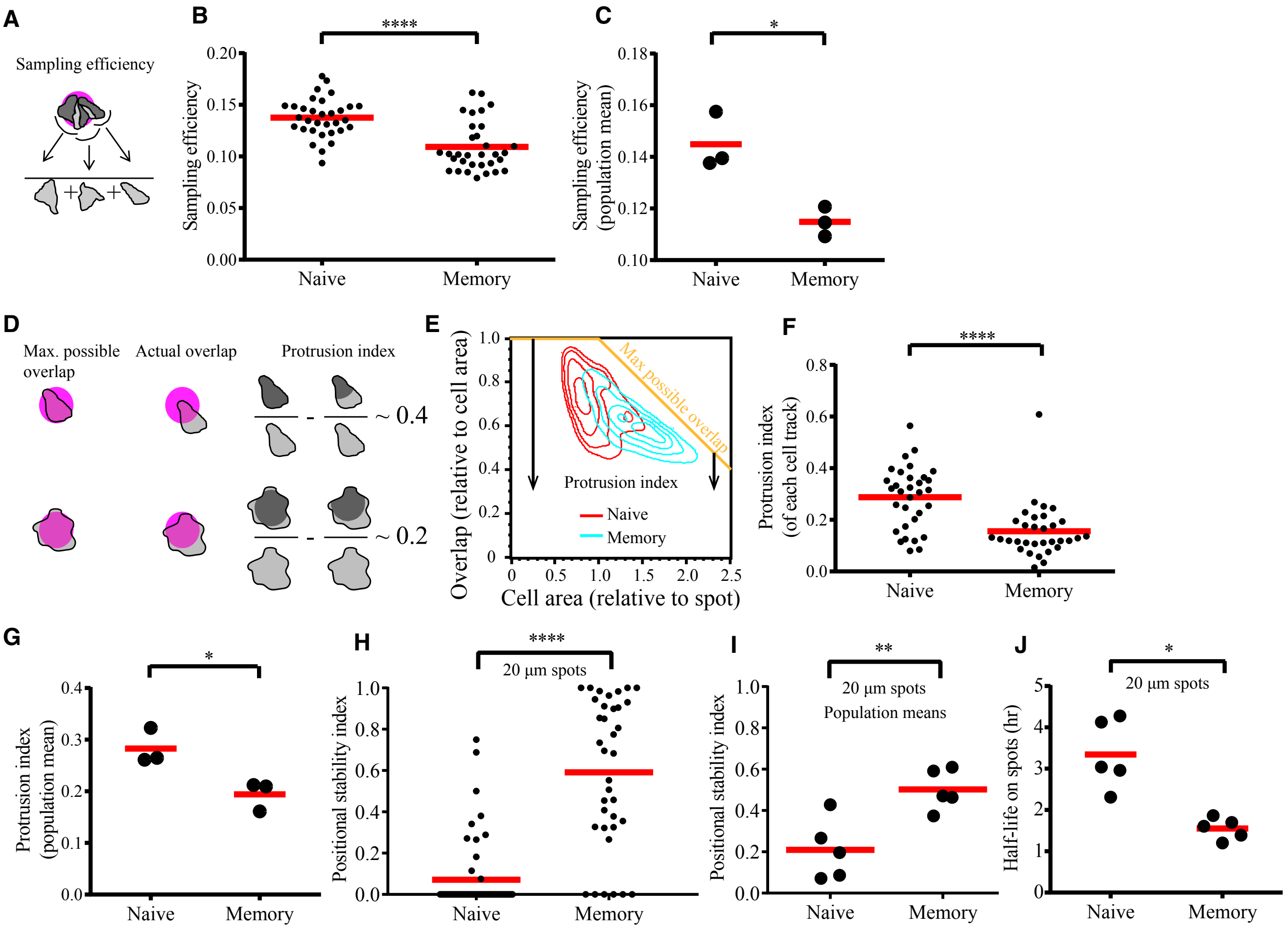

Figure 4. Human Naive CD8 T Cells Exhibit Durable Interactions Despite Kinapses on the Stimulatory Spots

(A–G) The motile tendency of human CD8 T cells engaged on 10-µm spots is quantified as sampling efficiency (A–C) and protrusion index (D–G).

(A) Sampling efficiency is defined as the fraction of unique pixels (denoted by darker shade of gray) over the total number of pixels (denoted by lighter shade of gray in the denominator) underneath the cell boundary within a certain duration of time (the illustration has 3 time steps). A cell with increased motile tendency should have a higher sampling efficiency.

(B and C) Naive cells have a higher sampling efficiency than memory cells when measured over 20 time steps. The same conclusion is drawn when 10 or 40 time steps were considered (data not shown). See also Figure S2.

(D) The protrusion index measures the fractional area of the cell that is outside of the stimulatory spot, which should increase with a more active search away from the stimulatory spot. The protrusion index of a cell at any instance is calculated by subtracting the fractional overlap from the maximum possible overlap (overlap is shown in dark gray). The maximum possible overlap reduces as the projected area of the cell increases beyond the size of the spot. Thus, considering the maximum possible overlap accounts for the increase in protrusion solely because of increased size and/or spreading of the cell.

(E) Contour map of the fractional overlap of naive (in red) and memory (in cyan) CD8 T cells. The contour plot was generated from data points representing each cell at each time point imaged. As the area of the cell grows beyond the area of the spot, the maximum possible overlap decreases linearly (orange trace). For any cell represented in the plot, the distance from the orange trace gives the protrusion index (shown by arrows for two cells).

(F and G) Naive cells have a higher protrusion index than memory cells.

(H and I) Naive human CD8 T cells exhibit a greater tendency for kinapses on 20-µm spots compared with memory cells. Note that only portions of tracks that did not contain any neighboring cell on the same spot were considered for this analysis to select for autonomous behavior.

(J) Human naive CD8 T cells exhibit a longer half-life of interaction on 20-µm-wide spots. This was the case even when the spots were completely occupied by crowding with 4–6 cells per spot instead of 1–3 cells per spot (data not shown).

The data points in (B), (F), and (H) represent individual cells or cell tracks from a particular donor, and those in (C), (G), and (I) represent population means from separate blood donors. Mean values of plotted data points are given as red horizontal bars.

their persistent movement always led them to form and retract protrusions off of the spot (Movie S4, for example). Overall, the naive cells showed a significantly lower positional stability index on 20-µm spots compared with the memory cells (Figures 4H and 4I). This was directly analogous to the behavior on uniformly coated surfaces (Figures 1E and 1F and 2E and 2H). The naive cells maintained the trend of a longer half-life of interaction (an average of 3.4 hr) despite continuous motility (Figure 4J). Further, human naive CD8 T cells exhibited durable interactions with mature monocyte-derived DCs embedded in collagen matrix

and presenting Okt3 that was captured via their Fc receptors (Movies S5 and S6). During these prolonged interactions, the naive cells exhibit continuous protrusions in different directions. One such event led to the naive cell shifting from one DC to another DC that comes into the neighborhood (Movie S5). This provides further proof that kinapse motility is intact during the prolonged interaction with DCs. Thus, our results demonstrate that naive T cells have prolonged interactions without forming a stable synapse and despite motile tendencies driven by the kinapse. We conclude that kinapses are not detrimental to the durability of interaction with spatially restricted stimulatory sites or with DCs.

DISCUSSION

Our *ex vivo* experiments mimic the two distinct scenarios of antigen presentation resulting from standard immunization regimens and allow the interaction mode to be determined quantitatively. Uniformly coated surfaces and SLBs emulate the scenario arising after peptide- or antigen-conjugated DEC-205 immunization, which results in spatially uniform antigen presentation by the DC network (Friedman et al., 2010; Hugues et al., 2004; Moreau et al., 2012; Sims et al., 2007). Kinapse motility observed *in situ* does not become a detrimental factor for prolonged signaling under these conditions because T cells remain in contact with antigen even as they move from DC to DC. Micropatterned surfaces mirror the scenario arising after DC immunization, in which antigen is presented in a spatially restricted manner by the emigrated DCs within the T cell zone. Taking advantage of the unique settings of optics and the substrate design in 2D, we have demonstrated that kinapse motility is intact on the stimulatory spots (Figure 4; Movies S3 and S4). However, these T cells exhibit durable interactions (Figures 3H and 3I and 4J), just as *in vivo* with DC immunization (Celli et al., 2007; Henrickson et al., 2008; Mempel et al., 2004). Motile tendency and crawling, similar to what we have observed on 10- and 20-μm stimulatory spots, can also be gleaned from the time-lapse images of T cell-dendritic cell interaction (T-DC) conjugates *in situ* (Figure S2E lists specific instances from the literature). This implies that the mode of engagement in those instances was via kinapses and not synapses. Thus, the extent of spatial distribution of antigen or anti-CD3ε dictates the spatial regime of naive T cell motility over several hours, either restricted to a single DC (or stimulatory spot) or across a larger area. This allows for reconciliation between the two seemingly incompatible features of T cell behavior: kinapse motility and durable interaction with DCs, which is required for the priming of naive T cells. We further note that our results do not rule out durable interactions in the synapse mode of interaction. Thus, durable interactions could arise both from synapses and kinapses.

Human memory T cells demonstrate a trend of lower half-life of interaction compared with their naive counterparts (Figures 3 and 4). We note that, after the memory cells leave a stimulatory spot, they latch onto another. Thus, the cooperative killing observed in antiviral responses could be a result of kinapses or serial, relatively short synapses (Halle et al., 2016). A shorter duration would allow the higher effector efficiency of synapses to be exploited without completely losing the possibility for local exploration inherent to kinapses (Beal et al., 2008; Huse et al., 2006). This is perhaps the reason for human memory CD8 T cells to employ stable synapses but limit their duration.

Our model system of stimulatory spots enabled us to demonstrate that T cells can engage in durable interactions without forming stable synapses. Because this model system mimics the main aspects of the *in situ* context under which naive T cells get primed (Bousso, 2008; Lindquist et al., 2004), we expect it to be of great utility in investigating the mechanistic underpinnings of T cell behavior varying from search for antigen and signal integration during priming to competition between T cells for antigen.

EXPERIMENTAL PROCEDURES

Ethics

Leukapheresis products (non-clinical and de-identified) from donor blood were used as a source of resting human T cells, which was exempt from institutional review board (IRB) review. The Non-Clinical Issue division of National Health Service approved the use of leukapheresis products at the University of Oxford (REC 11/H0711/7). All procedures and experiments involving mice were conducted at the New York University Medical Center and were approved by the Institutional Animal Care and Use Committee (protocol 150609-01).

Isolation of T Cell Subsets

Resting human T cell subsets were isolated from leukapheresis products using negative selection kits from STEMCELL Technologies. Mouse T cells were isolated either by negative selection or by sorting of relevant populations from B6 mice. Memory T cells were obtained from mice 30 days after infection with *Listeria monocytogenes*-expressing ovalbumin. OT-I T cells were isolated after adoptive transfer into congenic hosts.

Preparation of Stimulatory Surfaces

Labtek 8-well chambers (Nunc) were used for uniformly coated stimulatory surfaces. Micro-contact printing was carried out as described previously (Shen et al., 2008a). The repeating "spot" patterns spanned the entire length of the channel of the sticky-Slide $VI^{0.4}$ (Ibidi). The stamped coverslips were affixed to the sticky-Slide, and the channels were coated sequentially with CCL21 (10 μg/mL) and ICAM1 (3 μg/mL). SLBs presenting UCHT1 Fab′ and ICAM1 were assembled in sticky-Slide $VI^{0.4}$ channels essentially with the same approach as described before for the FSC2 Bioptechs flow chambers (Choudhuri et al., 2014; Dustin et al., 2007). The main difference is that the entire channel was filled with the liposome suspension to form a bilayer all along the channel.

Imaging

Cells were imaged using either a Zeiss LSM 510 or an Olympus FluoView FV1200 confocal microscope that was enclosed in an environment chamber (at 37°C) and operating under standard settings. These microscopes are equipped for collecting differential interference contrast (DIC) images used for detecting and tracking cells and for collecting reflection images used for ascertaining spreading or attachment. In some experiments, cells are labeled with CellTracker dyes (Life Technologies) to identify the T cells subsets. Calcium was imaged by the ratiometric method using Fluo4-acetylmethoxy ester. (AM) and Fura Red-AM (Wolf et al., 2015). The location of stimulatory spots was recorded using Alexa Fluor 647 conjugated to the stamped anti-CD3.

Image Analysis and Quantification of Various Metrics

The time-lapse images were pre-processed in ImageJ. Tracking and associated quantification were conducted using TIAM, a MATLAB-based toolset we have developed (Mayya et al., 2015). The code is available on Github (https://github.com/willieneis/TIAM). Bespoke functions and scripts were written in MATLAB for the calculation confinement score, positional stability index, sampling efficiency, protrusion index, and duration of interaction, with the spots using the output from TIAM. These functions are scripts also available on Github (https://github.com/uvmayya/kinapseVsDurability).

T Cell Activation Assays

Cell numbers equivalent to the number of stimulatory spots (~90,000) were introduced into the channel. The wells feeding the channel were simultaneously filled with additional medium. At various time points, the cells were collected using ice-cold PBS containing 0.5% BSA and 2 mM EDTA. These cells were appropriately assayed by flow cytometry for activation markers and cell division by dilution of Cell Trace Violet.

Statistical Methods

Statistical significance of difference in values, wherein a pair of values represents the T cell subsets of a donor, was calculated by paired t test. Statistical significance of difference in population behavior, wherein individual cells between subsets of a donor are compared, was calculated by Mann-Whitney *U* test. p values from two-tailed tests are denoted as follows in the figures: *$p < 0.05$, **$p < 0.01$, ***$p < 0.001$, ****$p < 0.0001$.

All experimental and analysis procedures are explained in further detail in the Supplemental Experimental Procedures.

SUPPLEMENTAL INFORMATION

Supplemental Information includes Supplemental Experimental Procedures, two figures, and six movies and can be found with this article online at https://doi.org/10.1016/j.celrep.2017.12.052.

ACKNOWLEDGMENTS

Supported by NIH grants PN2 EY016586 (to M.L.D. and L.C.K.) and R37 AI43542 (to M.L.D.), a Cancer Research Institute post-doctoral fellowship (to V.M.), Wellcome Trust Principal Research Fellowship 100262/Z/12/Z (to M.L.D.), funds from the Kennedy Trust for Rheumatology Research grant MSP121321 (to M.L.D. and the KIR Microscopy Facility), a Human Frontiers Science Program research grant RGP0033/2015 (to M.L.D.), and European Research Council grant AdG 670930-SYNECT (to M.L.D.). The assistance of the Light Microscopy and Flow Cytometry Core Facilities at the NYU Medical Center is also acknowledged. We thank A. Gondarenko for help with e-beam lithography, S. Valvo and J. Afrose for preparing key reagents, and A. Gerard for comments on the manuscript.

AUTHOR CONTRIBUTIONS

V.M. conceptualized the project, designed and performed experiments, analyzed the data, and co-wrote the manuscript. E.J. and L.C.K. designed the masters for micro-contact printing and participated in related method development. V.M., W.N., and C.H.W. developed the TIAM package. E.A.S. conducted the imaging of T-DC conjugates in collagen gels. C.G.P. participated in the development of metrics for positional stability. D.D. participated in method development related to Ibidi chambers and in lipid bilayer experiments. D.A.B. supported the animal experiments. M.L.D. supervised the research, facilitated collaboration, participated in method development, and co-wrote the manuscript. All authors discussed the results and approved the manuscript.

DECLARATION OF INTERESTS

The authors declare no competing interests.

Received: May 16, 2017
Revised: October 2, 2017
Accepted: December 14, 2017
Published: January 9, 2018

REFERENCES

Adachi, K., and Davis, M.M. (2011). T-cell receptor ligation induces distinct signaling pathways in naive vs. antigen-experienced T cells. Proc. Natl. Acad. Sci. USA *108*, 1549–1554.

Beal, A.M., Anikeeva, N., Varma, R., Cameron, T.O., Norris, P.J., Dustin, M.L., and Sykulev, Y. (2008). Protein kinase C theta regulates stability of the peripheral adhesion ring junction and contributes to the sensitivity of target cell lysis by CTL. J. Immunol. *181*, 4815–4824.

Bousso, P. (2008). T-cell activation by dendritic cells in the lymph node: lessons from the movies. Nat. Rev. Immunol. *8*, 675–684.

Celli, S., Lemaître, F., and Bousso, P. (2007). Real-time manipulation of T cell-dendritic cell interactions in vivo reveals the importance of prolonged contacts for CD4+ T cell activation. Immunity *27*, 625–634.

Choudhuri, K., Llodrá, J., Roth, E.W., Tsai, J., Gordo, S., Wucherpfennig, K.W., Kam, L.C., Stokes, D.L., and Dustin, M.L. (2014). Polarized release of T-cell-receptor-enriched microvesicles at the immunological synapse. Nature *507*, 118–123.

Davis, D.M. (2009). Mechanisms and functions for the duration of intercellular contacts made by lymphocytes. Nat. Rev. Immunol. *9*, 543–555.

Doh, J., and Irvine, D.J. (2006). Immunological synapse arrays: patterned protein surfaces that modulate immunological synapse structure formation in T cells. Proc. Natl. Acad. Sci. USA *103*, 5700–5705.

Dustin, M.L. (2007). Cell adhesion molecules and actin cytoskeleton at immune synapses and kinapses. Curr. Opin. Cell Biol. *19*, 529–533.

Dustin, M.L., Bromley, S.K., Kan, Z., Peterson, D.A., and Unanue, E.R. (1997). Antigen receptor engagement delivers a stop signal to migrating T lymphocytes. Proc. Natl. Acad. Sci. USA *94*, 3909–3913.

Dustin, M.L., Starr, T., Varma, R., and Thomas, V.K. (2007). Supported planar bilayers for study of the immunological synapse. Curr. Protoc. Immunol. *Chapter 18*, Unit 18.13.

Friedman, R.S., Beemiller, P., Sorensen, C.M., Jacobelli, J., and Krummel, M.F. (2010). Real-time analysis of T cell receptors in naive cells in vitro and in vivo reveals flexibility in synapse and signaling dynamics. J. Exp. Med. *207*, 2733–2749.

Gunzer, M., Schäfer, A., Borgmann, S., Grabbe, S., Zänker, K.S., Bröcker, E.B., Kämpgen, E., and Friedl, P. (2000). Antigen presentation in extracellular matrix: interactions of T cells with dendritic cells are dynamic, short lived, and sequential. Immunity *13*, 323–332.

Halle, S., Keyser, K.A., Stahl, F.R., Busche, A., Marquardt, A., Zheng, X., Galla, M., Heissmeyer, V., Heller, K., Boelter, J., et al. (2016). In Vivo Killing Capacity of Cytotoxic T Cells Is Limited and Involves Dynamic Interactions and T Cell Cooperativity. Immunity *44*, 233–245.

Henrickson, S.E., Mempel, T.R., Mazo, I.B., Liu, B., Artyomov, M.N., Zheng, H., Peixoto, A., Flynn, M.P., Senman, B., Junt, T., et al. (2008). T cell sensing of antigen dose governs interactive behavior with dendritic cells and sets a threshold for T cell activation. Nat. Immunol. *9*, 282–291.

Hugues, S., Fetler, L., Bonifaz, L., Helft, J., Amblard, F., and Amigorena, S. (2004). Distinct T cell dynamics in lymph nodes during the induction of tolerance and immunity. Nat. Immunol. *5*, 1235–1242.

Huse, M., Lillemeier, B.F., Kuhns, M.S., Chen, D.S., and Davis, M.M. (2006). T cells use two directionally distinct pathways for cytokine secretion. Nat. Immunol. *7*, 247–255.

Iezzi, G., Karjalainen, K., and Lanzavecchia, A. (1998). The duration of antigenic stimulation determines the fate of naive and effector T cells. Immunity *8*, 89–95.

Lindquist, R.L., Shakhar, G., Dudziak, D., Wardemann, H., Eisenreich, T., Dustin, M.L., and Nussenzweig, M.C. (2004). Visualizing dendritic cell networks in vivo. Nat. Immunol. *5*, 1243–1250.

Mayya, V., Neiswanger, W., Medina, R., Wiggins, C.H., and Dustin, M.L. (2015). Integrative analysis of T cell motility from multi-channel microscopy data using TIAM. J. Immunol. Methods *416*, 84–93.

Mempel, T.R., Henrickson, S.E., and Von Andrian, U.H. (2004). T-cell priming by dendritic cells in lymph nodes occurs in three distinct phases. Nature *427*, 154–159.

Miller, M.J., Safrina, O., Parker, I., and Cahalan, M.D. (2004). Imaging the single cell dynamics of CD4+ T cell activation by dendritic cells in lymph nodes. J. Exp. Med. *200*, 847–856.

Moreau, H.D., and Bousso, P. (2014). Visualizing how T cells collect activation signals in vivo. Curr. Opin. Immunol. *26*, 56–62.

Moreau, H.D., Lemaître, F., Terriac, E., Azar, G., Piel, M., Lennon-Dumenil, A.M., and Bousso, P. (2012). Dynamic in situ cytometry uncovers T cell receptor signaling during immunological synapses and kinapses in vivo. Immunity *37*, 351–363.

Moreau, H.D., Lemaître, F., Garrod, K.R., Garcia, Z., Lennon-Duménil, A.M., and Bousso, P. (2015). Signal strength regulates antigen-mediated T-cell deceleration by distinct mechanisms to promote local exploration or arrest. Proc. Natl. Acad. Sci. USA *112*, 12151–12156.

Parsey, M.V., and Lewis, G.K. (1993). Actin polymerization and pseudopod reorganization accompany anti-CD3-induced growth arrest in Jurkat T cells. J. Immunol. *151*, 1881–1893.

Shen, K., Qi, J., and Kam, L.C. (2008a). Microcontact printing of proteins for cell biology. J. Vis. Exp. *22*, 1065.

Shen, K., Thomas, V.K., Dustin, M.L., and Kam, L.C. (2008b). Micropatterning of costimulatory ligands enhances CD4+ T cell function. Proc. Natl. Acad. Sci. USA *105*, 7791–7796.

Sims, T.N., Soos, T.J., Xenias, H.S., Dubin-Thaler, B., Hofman, J.M., Waite, J.C., Cameron, T.O., Thomas, V.K., Varma, R., Wiggins, C.H., et al. (2007). Opposing effects of PKCtheta and WASp on symmetry breaking and relocation of the immunological synapse. Cell *129*, 773–785.

Simson, R., Sheets, E.D., and Jacobson, K. (1995). Detection of temporary lateral confinement of membrane proteins using single-particle tracking analysis. Biophys. J. *69*, 989–993.

Sung, J.H., Zhang, H., Moseman, E.A., Alvarez, D., Iannacone, M., Henrickson, S.E., de la Torre, J.C., Groom, J.R., Luster, A.D., and von Andrian, U.H. (2012). Chemokine guidance of central memory T cells is critical for antiviral recall responses in lymph nodes. Cell *150*, 1249–1263.

Wolf, I.M., Diercks, B.P., Gattkowski, E., Czarniak, F., Kempski, J., Werner, R., Schetelig, D., Mittrücker, H.W., Schumacher, V., von Osten, M., et al. (2015). Frontrunners of T cell activation: Initial, localized Ca2+ signals mediated by NAADP and the type 1 ryanodine receptor. Sci. Signal. *8*, ra102.

Woolf, E., Grigorova, I., Sagiv, A., Grabovsky, V., Feigelson, S.W., Shulman, Z., Hartmann, T., Sixt, M., Cyster, J.G., and Alon, R. (2007). Lymph node chemokines promote sustained T lymphocyte motility without triggering stable integrin adhesiveness in the absence of shear forces. Nat. Immunol. *8*, 1076–1085.

Zanin-Zhorov, A., Ding, Y., Kumari, S., Attur, M., Hippen, K.L., Brown, M., Blazar, B.R., Abramson, S.B., Lafaille, J.J., and Dustin, M.L. (2010). Protein kinase C-theta mediates negative feedback on regulatory T cell function. Science *328*, 372–376.

Cell Reports, Volume *22*

Supplemental Information

Durable Interactions of T Cells with T Cell Receptor Stimuli in the Absence of a Stable Immunological Synapse

Viveka Mayya, Edward Judokusumo, Enas Abu Shah, Christopher G. Peel, Willie Neiswanger, David Depoil, David A. Blair, Chris H. Wiggins, Lance C. Kam, and Michael L. Dustin

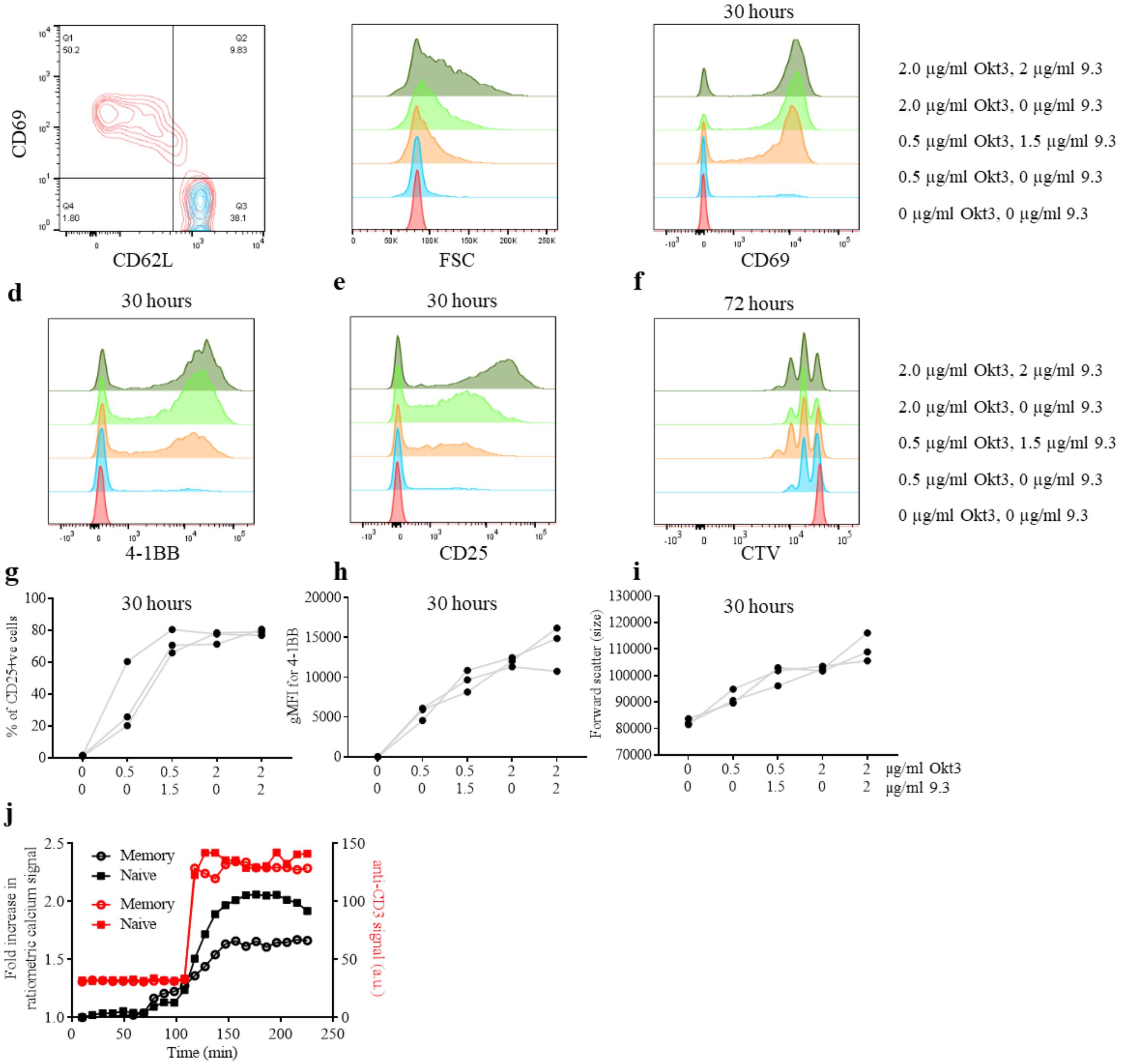

Figure S1: (Related to Figure 3). Priming and activation of human naïve CD8 T cells on stimulatory spots. a-e) Activation status of primed naïve CD8 T cells on stimulatory spots stamped with varying amounts of Okt3 and 9.3 antibodies. The stained surface markers are indicated as well as the duration of interaction with the spots. f) Proliferation of naïve CD8 T cells assayed by dilution of Cell Trace Violet due to cell-division. g to i) Quantification of cytometric information summarised for 3 donors. j) Memory CD8 T cells flux less calcium compared to the naïve cells when they arrest on the stimulatory spots.

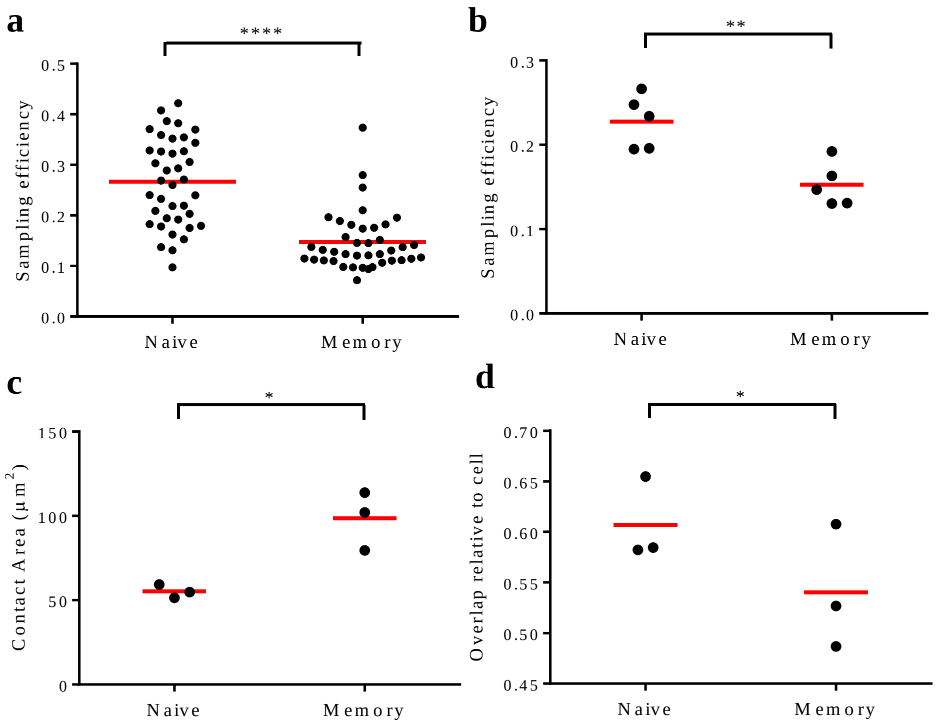

e

Behavior	Study	Name
Motile tendency on DC	Chemokine guidance of central memory cells is critical for anti-viral responses in lymph nodes (PMID: 22980984).	Movie S1
Motile tendency on DC	T cell sensing of antigen dose governs interactive behaviour with Dendritic cells and sets a threshold for T cell activation (PMID: 22980984).	Supplementary video 6, 7, 9
Crawling on DCs	Dynamics of CD8+ T cell priming by Dendritic cells in intact lymph nodes (PMID: 12730692)	Supplementary video 3
Motile tendency on DC	Imaging the single cell dynamics of CD4+ T cells by dendritic cells in lymph nodes (PMID: 15466619)	Video S5, S7

Figure S2: (Related to Figure 4). a and b) Sampling efficiency of naive and memory human CD8 T cells on anti-CD3 and ICAM1 coated glass. Sampling efficiency is defined as the fraction of unique pixels over the total number of pixels underneath the cell-boundary within certain duration of time (20 time-steps or 10 minutes in this case, as in Figure 4b and 4c). A cell with increased motile tendency should have higher sampling efficiency. Data-points in panel a represent individual tracks of cells from a particular donor and in panel b they represent population means from separate blood donors. Statistical significance of difference in population behavior was calculated by Mann-Whitney U-test (in a). Statistical significance of difference in mean values was calculated by paired t-test (in b). c and d) Memory human CD8 T cells have

larger contact area but lesser fractional overlap with the 10 µm stimulatory spots. Mean values from a population of cells from each of the three donors is shown. Statistical significance of difference in mean values was calculated by paired t-test. * for $p \leq 0.05$, ** for $p < 0.01$ and **** for $p < 0.0001$. e) Source of instances in the literature wherein motile tendency of naïve T cells conjugated to antigen-bearing DCs is visible. This has also been noticed by others (Celli et al., 2008).

Description of the videos

Video 1: Behaviour of human CD8 T cells on uniform stimulatory surfaces (Related to Figure 1). Time-lapse video of the motility behaviour of CMRA-labelled naïve (in red) and CMFDA-labelled memory (in green) human CD8 T cells on immobilized OKT3 and ICAM1. Field of imaging was 131.84 µm in size. Progression of time is also noted in the video.

Video 2: Behaviour of human CD8 T cells on cell-sized stimulatory spots (Related to Figure 3). Side-by-side comparison of durability of interaction of naïve (on left) and memory (on right) human CD8 T cells with 10µm stimulatory spots. Spots are shown in magenta. In the upper half, a positive mask of the dilated spot and contact foot-print was applied to the DIC images so that only the cells engaged on the spots were tracked by TIAM. Masked DIC images used for the video here helps with avoiding distraction from motile cells that pass by the spots. In the lower half, the same dataset is shown without the mask. There was some advection in the channel containing naïve cells, as a results of which motile cells have a bias in their movement towards the top of the field. Nonetheless, scanning motility of T cells can be appreciated in the lower half of the video. It is to be noted that advection does not impact durability of interaction. Tracked positions are overlaid as small yellow squares on the masked DIC images. A bigger yellow square flashes to indicate the termination of the track, in other words, signifies dissolution of the synapse and exit from the spot. Memory cells exhibit more such events, implying reduced half-life of interaction on antigenic spots. There were some technical challenges in calculating the half-life of interaction for memory cells, which are detailed in the Image Analysis sub-section of Supplementary Methods. The memory cells that leave a spot, typically engage with another neighboring spot. This can be appreciated by the sizeable number of attached memory cells on spots that were not tracked, as their tracks did not begin at the initially considered frame.

Video 3: Kinapse behaviour highlighted for a single naïve CD8 T cell on a 10µm spot (Related to Figure 4).Time-lapse video of a single naïve CD8 T cell showing prolonged engagement (3 hours) with a 10µm OKT3 spot despite constant generation of protrusions away from the spot. Occasionally, the cell also forms a nascent uropod and a dominant, single protrusion at the opposite end, both of which are transient. The cell is shown as an overlay of DIC and IRM to provide a darker contrast and thus distinguish the concerned cell from other passing-by cells that transiently engage the spot without a juxtaposed contact. Outline of the spot and the cell boundary are also provided on the right side to aid visualization.

Video 4: Kinapse behaviour highlighted for a single naïve CD8 T cell on a 20μm spot (Related to Figure 4).Time-lapse video of a single naïve CD8 T cell showing prolonged engagement (>2 hours) with a 20μm OKT3 spot despite constant kinapse motility along the circumference of the spot. As in video 3, the cell is shown as an overlay of DIC and IRM to provide a darker contrast and thus distinguish the concerned cell from other passing-by cells that transiently engage the spot without a juxtaposed contact. Outline of the spot and the cell boundary are also provided on the right side to aid visualization. The motility of the T cell is seemingly confined or dictated by the boundary of the spot.

Video 5 and 6: Naïve CD8 T cells in prolonged engagement with DCs while exhibiting kinapse behaviour (Related to Figure 4). 3D **t**ime-lapse video of human naïve CD8 T cells (in green) engaging in prolonged interaction with mature monocyte derived DCs (in magenta) that were loaded with Okt3 via their Fc Receptors and embedded in collagen matrix. Motile tendency and protrusive behaviour persists throughout the 1.5 hours of engagement that was captured. We did not observe naïve cells disengaging from the DCs within the 1.5 hours of observation. The migratory movement of other cells in the field was primarily due to CCL19 that was added to the collagen matrix. In video 5, the engaged T cell shifts from one DC to another DC that comes into the vicinity. This implies kinapse mode of interaction with the DCs. In video 6 multiple naïve cells can be seen jostling with motile tendency, yet they stay engaged with the DC. Observations are representative of two separate experiments.

Detailed experimental procedures

Isolation of human T cells

Resting T cells were isolated from leukapheresis products (non-clinical) obtained either from the New York Blood Center (for experiments conducted at the Skirball Institute, NYU Medical Center, New York, USA) or the National Health Service Blood and Transplant Center at the John Radcliffe Hospital (for experiments conducted at the Kennedy Institute of Rheumatology, University of Oxford, Oxford, UK). Total CD8 or CD4 population was enriched using the Rosette Sep (Stemcell Technologies) approach. Naïve and memory cells were isolated by negative selection using the respective EasySep Enrichment Kits (Stemcell Technologies). In some instances memory CD4 cells were isolated by positive selection using CD45RO

microbeads (Miltenyi Biotec) with the flow-through from the LD column used as the naïve CD4 population. Cytometric assessment using CD62L (clone DREG-56, from BD Biosciences) and CD45RO (clone UCHL1, from BD) as surface markers indicated that naïve and memory cells were typically >90% in purity. Cells were cultured for a maximum of 5 days at a density of 3 million/ml in phenol-red free RPMI medium supplemented with 25 mM HEPES, 2 mM glutamine, 1 mM sodium pyruvate, and 10% fetal bovine serum (also used as imaging medium) until imaging.

Isolation of murine CD8 T cells

Cells were obtained from 8-14 week old C57BL/6 (B6) mice or their congenic CD45.1 (commonly referred as SJL mice) counterparts housed in specific pathogen-free conditions. These mice were obtained either from Jackson Laboratory or the NCI mouse repository. OT-I TCR transgenic mice deficient in recombinant-activating gene 1 (RAG1) were bred in-house. All procedures and experiments involving mice were conducted at the Skirball Institute and were approved by the Institutional Animal Care and Use Committee. 1×10^5 OT-I naïve T cells were adoptively transferred into SJL mice by tail vein injection. These SJL mice were infected with 5×10^3 colony-forming units of *Listeria monocytogenes* expressing ovalbumin (Lm-ova) by tail vein injection. The infected mice were used as a source of memory OT-I T cells 30-40 days later. Naïve OT-I T cells were obtained from naïve SJL recipient mice, 1-2 days after transfer of 1×10^6 cells, instead of being directly used. This was done to subject both naïve and memory OT-I T cells to the same procedure of isolation and handling. Polyclonal memory CD8 T cells (defined as $CD44^{hi}$) were obtained from B6 mice that were infected with Lm-ova 30-40 days prior to use. CD44-ve CD8 T cells from the same mice were used as polyclonal naïve CD8 T cells. On the

day of the isolation, the CD8 T cells were first enriched from splenocytes by negative selection using the Dynabeads Untouched Mouse CD8 Cells Kit (Life Technologies/Thermo Fisher). The desired cell populations were then isolated by sorting into cold fetal bovine serum using FACSAria (BD Biosciences). OT1-I T cells were selected as $CD4^-$, $B220^-$, $NK1.1^-$, $MHC\text{-}II^-$, $CD8^+$ and $CD45.2^+$ cells. Polyclonal CD8 T cells were selected as $CD4^-$, $B220^-$, $NK1.1^-$, $MHC\text{-}II^-$ cells with the status of CD44 staining defining the memory and naïve populations as mentioned above. The cells were kept on ice for a maximum of six hours and warmed to 37 °C in imaging medium (see earlier section) for 20 minutes, just before introducing them into the imaging chamber.

The antibodies used for sorting the naïve and memory cells are as follows: CD4 (clone RM4-4, Biolegend), B220 (clone RA3-6B2, Biolegend), NK1.1 (clone PK136, eBioscience), MHC-II (clone M5/114.15.2, eBioscience), CD45.1 (clone A20, Biolegend), CD45.2 (clone 104, eBioscience), CD8α (clone 53-6.7, eBioscience), CD44 (clone IM7, Biolegend).

Preparation of stimulatory surfaces

Uniform coated chambers: Uniform coated surfaces present spatially unlimited ligands adsorbed and immobilized from solution-phase. #1 or #1.5 Labtek 8-well chambers (Nunc) with cover-glass bottom were coated first with 3 µg/ml of recombinant CCL21 (from R&D systems or Peprotech) in 200 µl of phosphate-buffered saline (PBS, pH 7.2) for one hour and then with a solution containing both ICAM1 (ectodomain of murine ICAM1 produced in S2 insect cells and used at 2µg/ml) and anti-CD3 (Okt3 for human cells or 145-2C11 for murine cells, from Ebioscience or BioXcell) in PBS for three hours at 37 °C. In some experiments coating with

CCL21 was omitted. Okt3 was used at 2 μg/ml for a complete response, i.e. for nearly all human naïve CD8 T cells to attach or at 0.05 μg/ml for threshold density of anti-CD3, below which negligible number of human naïve CD8 T cells attach. Anti-CD28 (clone 9.3, BioXcell) and anti-CD8 (Okt8, eBioscience) were used at 1 μg/ml along with ICAM1 and Okt3.

Micro-contact printed chambers: Micro-patterned surfaces presenting spatially limited features of activating and adhesive ligands were prepared by micro-contact printing (Shen et al., 2008a; Shen et al., 2008b). Briefly, topological masters were developed on silicon wafers by patterning a spin-coated layer of poly-methyl methacrylate (PMMA) using electron beam lithography. The following two patterns of circles were defined: 1) 10 μm in diameter, spaced 30 μm center-to-center on a square grid, and; 2) 20 μm in diameter, spaced 50 μm. These patterns were repeated to cover the entire length of the channel of the sticky-Slide $VI^{0.4}$ (Ibidi). The master was then silanized for multiple casting of polydimethylsiloxane (PDMS) elastomer stamps. Sylgard 184 (Dow Corning) PDMS was used for these stamps, mixing 1 part curing agent for 7 parts of the elastomer by mass. Rectangular stamps of PDMS were coated with dye-labeled (using N-Hydroxysuccinimide chemistry) activating antibodies to CD3 (2 μg/ml of Okt3 or 5 μg/ml of 145-2C11) in 150 μl of PBS for one hour. The blocks were then rinsed extensively in PBS, PBS with 0.05% Tween-20 and finally in MilliQ-grade water followed by gentle drying with N2 to remove droplets of water. Borosilicate coverslips (either 24 mm × 40 mm Fisherbrand Cover Glass from Fisher Scientific or 25 mm ×75 mm D 263 M Schott Glass from Ibidi) were pre-cleaned by rinsing sequentially in MilliQ-grade water (10 minutes), 70% ethanol (1 minute) and running deionized water and then baked at 400 °C for 10 hours prior to use as a substrate for micro-contact printing. Anti-CD3 coated PDMS blocks were stamped onto the coverslips for 5

minutes under ~20 g of load for consistency. The patterned coverslip was then affixed to the sticky-Slide VI$^{0.4}$ (Ibidi) and washed sequentially with MilliQ-grade water and PBS. The channels were then coated with 13.5 µg/ml of CCL21 in 30 µl for one hour and then 3 µg/ml of ICAM1 in 180 µl for three hours. The quality and consistency of stamping were confirmed in every case by confocal microscopy, taking advantage of conjugated Alexa Fluor 647 (Molecular Probes) and periodically by macroscopic infra-red imaging (Odyssey Imaging System) of the entire chamber, taking advantage of conjugated IRDye 680LT (LI-COR Biosciences).

Supported Lipid Bilayer chambers: Supported Lipid Bilayers (SLBs) present spatially unlimited and laterally mobile ligands in native state of orientation and structure. Assembly of SLBs presenting UCHT1 Fab' and ICAM1 was conducted essentially as described before, with minor modifications (Choudhuri et al., 2014; Dustin et al., 2007). Small unilamellar liposomes were prepared by extrusion (from Avestin; 100 nm pore size filter) of reconstituted phospholipids (from Aventi Polar Lipids). The molar % of lipids in the liposomes was as follows: 97.5% DOPC, 2.5% DOGS-NTA, and 0.004% DOPE-cap-biotin. The molar fraction of DOPE-cap-biotin was titrated to provide ~30 molecules/µm^2 of UCHT1 Fab'. Bilayers were formed by filling the channels created in the sticky-Slide VI$^{0.4}$ (Ibidi) using 30 µl of liposomes after affixing borosilicate cover-glass (SCHOTT NEXTERION) cleaned with peroxidated H_2SO_4 (piranha solution). ICAM1 was presented at a surface density of ~200 molecules/µm^2.

Cytometry to profile the activation status of naïve T cells

Equivalent numbers of cells as the number of stimulatory spots (~90,000) were introduced into the channel in 30 µl of culture medium. The wells feeding the channel were simultaneously filled

with additional medium using a multi-channel pipette. This prevents displacement of cells from the channel. At various time-points, the all the cells in the channel were collected using ice-cold PBS containing 0.5% BSA and 2mM EDTA, which was found to be effective in dislodging attached cells. These cells were appropriately assayed by flow cytometry for activation markers and cell division by dilution of Cell Trace Violet (CTV). Cells were labelled with 0.3 µM of CTV a priori for measurement of proliferation. The following antibodies were used against the activation markers: CD69 (clone FN50, Biolegend), CD62L (DREG-56, Biolegend), 4-1BB (4B4-1, Biolegend), and CD25 (MA25-1, Biolegend).

Imaging

Imaging in the Labtek 8-well chambers was done with 1 million cells/ml (200 µl) and imaging in the Ibidi sticky-Slide VI$^{0.4}$ channels was done with 3-4 million cells/ml (150 µl) for 10 µm spots and either 5-6 million cells/ml (for crowding on the spots) or 1-2 million cells/ml for 20 µm spots. The cells were pre-treated with inhibitors of PKCθ/α for 20 minutes before using them in the specified experiments in the presence of the inhibitors. Myristoylated pseudosubstrate peptides of PKCα and PKCθ (20 µM; from Calbiochem) inhibit the respective kinases by binding to the active site in a competitive manner. C20 (1 µM) is a compound from Boehringer Ingelheim that acts on PKCθ by non-competitive binding to the active site.

Naïve and memory T cells were imaged together in the same chamber in certain experiments. The cells were differentially labelled with CellTracker dyes CMFDA (at 50 or 100 nM) and CMRA (at 200 or 250 nM) for these experiments. The labelling was in PBS at room temperature for 15 minutes. Cells were washed in PBS before and after the dye labelling. The labelled cells

were used after 2-3 hours and within two days for the experiments. The results were consistent with those obtained without labelling or when the dyes were swapped between the cell types. Calcium imaging was performed by the ratiometric method using the Fluo4-AM (at 3 µM) and Fura Red-AM (at 6 µM) dyes (Wolf et al., 2015). Washed cells were incubated with the dyes for 30 minutes in serum free RPMI medium at 37 °C, washed and further rested for 30 minutes in the imaging medium at 37 °C. Dye-loaded cells were used for imaging within 3 hours.

Cells were imaged using either a Zeiss LSM 510 or an Olympus FluoView FV1200 confocal microscope that was enclosed in an environment chamber (at 37 °C) and operating under standard settings. 40x Plan Neofluar oil immersion objective (1.3 NA) was used on the Zeiss LSM510 and 30x Super Apochromat silicone oil immersion objective (1.05 NA) was used on the Olympus Fluoview FV1200 microscope. Both these lenses are compatible with Differential Interference Contrast (DIC) microscopy. Both microscopes were equipped with a DIC prism to collect DIC images via the transmitted light. Reflected light from the incident laser (typically 543 nm on LSM510 and 635 nm on FV1200) is captured for interference reflection microscopy (IRM). This is facilitated by the flexibility in the choice of dichroics available on these microscopes. Reflected light is captured to record interference that occurs between light reflected from the cover-slip and the closely apposing cell membrane. This provides information on adhesion, spreading, and protrusions of cells. The same laser is used for both IRM and DIC with pixel dwell time of 4 or 8 µsec to minimize photo-toxicity and the build-up of free radicals over hours of acquisition. 8- or 12-bit images of 512-by-512 pixel size were collected at 1x zoom, with an interval of 30 seconds in between. The positions of the micro-contact printed stimulatory spots are recorded just prior to the commencement of the live imaging and not as part of time-

lapse acquisition. Fluorescence channels were included for time-lapse acquisition when the cells were labelled with CellTracker dyes or calcium dyes.

Image analysis

Image analysis and quantification was conducted using TIAM (Tool for Integrative Analysis of Motility), a MATLAB based toolset that we have developed (Mayya et al., 2015). Cells are detected and tracked using the transmitted light images (DIC here). Cell positions are then used to perform local segmentation to features of cells from every image channel. The features include the segmented image itself, outline of the cell in a particular channel and the extracted information such as morphological polarity, contact area and mean fluorescence intensity of the cell. These features and motility-related parameters are stored for every cell position of a track in MATLAB .mat files. We have previously assessed the accuracy of tracking and extracting information using TIAM from similar time-lapse datasets (Mayya et al., 2015). The graphic-user interface of TIAM allows for ensuring that the chosen detection parameters result in good detection. Similarly, individual tracks can be assessed in video-mode. Further, outlines of cells in different channels can be stored and overlaid on the actual images in ImageJ. These visual aids were used when deemed necessary.

IRM+ve parts of tracks were selected prior to the calculation of arrest coefficient. A threshold speed of 0.5 µm/min was used for calculating the arrest coefficient. This threshold was chosen based on the observation that most of the fully sessile human memory CD8 T cells (i.e. with <10 µm net displacement after 2 hours) had an average speed of < 0.5 µm/min. It is to be noted that the speed referred here is not the raw instantaneous speed, but rather a 'smoothed' instantaneous

speed by considering displacement over 5 time-steps centered on the time-point of interest. The smoothed speed was considered to reduce the effects of 'wiggle' in the positioning of the centroid that is influenced by protrusive dynamics of the cells.

Pre-processing of images in ImageJ: Microscopy data files are opened as hyperstacks in ImageJ and then converted to 8-bit depth, if necessary. 'Remove Outliers' routine in ImageJ was used to remove constructive interference patterns in IRM images and replace them with pixel values from the neighbourhood. Then the 'Enhance contrast' routine was used to normalize (min-max) the intensity distribution. IRM is very sensitive to focus and planarity drifts, which can lead to spatiotemporal changes in background and foreground intensity. While local segmentation by TIAM is robust to most of these changes, abrupt spatial gradients in background can lead to faulty segmentation. If abrupt spatial gradients were observed, 'bandpass FFT filter' operation was conducted to minimize gradients. The hyperstacks are stored as tiff image series in separate folder for analysis in TIAM.

A positive mask of the dilated spot and contact foot-print was applied to the DIC images so that only the cells engaged on the spots were tracked by TIAM for half-life measurements. Global segmentation was necessary for creating the positive masks. Mask of the spots was created by segmentation using the 'Default' thresholding algorithm in ImageJ. IRM images were processed as described above and the 'Default' thresholding algorithm was used for creating mask of contact foot-prints. Both the masks were dilated by multiple pixels before creating a combined mask by the 'AND' operation between the masks. Such a mask was also applied to the DIC images to quantify the arrest coefficient of cells engaged on 20 µm spots.

A positive mask of the cells was created using the ‘Default’ thresholding algorirthm in ImageJ on median-filtered image of cells in the Fura-red channel. The positive mask was applied on the ratio image (Fluo-4 over Fura-Red) in 32-bit. It was then converted to 8-bit without loss of information. The ratio image was considered as a fluorescence image by TIAM.

Determination of confinement and positional stability from tracks of cells:

Confinement score is same as probability level L defined previously for the analysis of single particle tracks of membrane proteins (Simson et al., 1995). L was mathematically defined as

$$L = \begin{cases} -\log(\psi) - 1, & when\ \psi \le 0.1 \\ 0, & when\ \psi > 0.1 \end{cases}$$

Wherein,

$$log\,\psi = 0.2048 - 2.5117Dt/R^2$$

ψ represents the probability that a particle with Brownian diffusion coefficient of D can stay within a distance of R within time t. L was calculated for short track-segments of varying lengths (4 to 10 frames, which defines t) in a sliding window fashion. D was set at 2 μm^2/frame (same as 4 μm^2/min). Largest displacement within the track-segment defines R. Finally, for every position in the track, L is averaged over all track-segments containing that position. Thus, periods with high L represent periods of relative confinement. It is to be noted that value of D changes the inferred values of L, hence the confinement being relative to D. L>3 represents a probability of <0.017 that the relative confinement is by random chance.

Once periods of relative confinement are defined, the algorithm calculates the positional spread within each period. Positional spread is defined as R^2/t, where t is the period of relative

confinement in number of frames and R is the diameter of the confined zone in µm. Diameter of confined zone was calculated based on the average of top-10 most distant pairings of points in the confined zone. A value of <0.666 for R^2/t was found to represent positional stability and all points within the period of relative confinement were assigned the stable state, i.e. value of 1, with default representing kinapse state (value of 0). If the positional spread was above this threshold, then the period of relative confinement was trimmed from the ends until the narrowed period of positional stability was found. If the period of relative confinement was less than 10 frames (5 minutes), it was not considered for positional stability.

Assignment of stable state was found to be largely robust to varying the maximum length of the track segment up to 15 (from 10) and considering a threshold value of 4 for L (from 3). Similarly higher values of D also provided essentially the same results.

Temporal alignment of tracks for the analysis of calcium flux when arresting on the stimulatory spots: Fluorescence intensity of the spots was reported by TIAM only when the local segmentation by TIAM was successful and when the centroid of the cell and the centroid of the segmented area of the spot are less than 1.5 µm apart. This gives an abrupt increase in the spot intensity in the track trace, making manual temporal alignment of multiple tracks feasible. Temporal alignment captures the average behaviour from many cells as they decelerate, arrest and spread onto the stimulatory spot.

Calculation of half-life of interaction on stimulatory spots: Cells that are attached on the spots are the only cells that are present in the finalized images as a result of masking, and

thus are the only cells that are tracked. During the tracking by TIAM, a track gets terminated if the cell leaves the spot, in other words, disappears from the masked image. ‘Survival’ plot of the tracks then provides off-rate and half-life measurements based on approximation of first-order kinetics.

In the benchmark dataset shown in Video 2, manual assessment of exit events revealed that 1 out of the 6 events called by TIAM were wrong for human naïve CD8 T cells and 8 out of 25 events called by TIAM were wrong in the case of human memory CD8 T cells. While the error rate is higher in the case of memory cells, the conclusions are not changed by the higher error. Several factors contribute to higher error in the case of memory cells, which cannot fully be addressed due to trade-off effects in the choice of parameters for detection and tracking: 1) Increased spreading of memory cells necessitates a larger (dilated) area of mask around the spots. However, a larger area also leads to inclusion, detection and tracking of motile cells that are transiently passing by the spots. This can lead to termination of an attached track due to a ‘track-switching’ error. 2) Because of less durable interactions memory cells leave the spot and ‘search’ for another spot to occupy. Thus, there are more instances of memory cells competing among themselves, jostling for space and occupancy on the spot. This also increases the chance of track-switching errors. 3) Increased instances of two or more cells on the same spot necessitate keeping the minimum separation distance allowed between cells to be as low as possible. However, this also increases multi-detection of a single cell.

Quantification of motile tendencies on 10 µm-wide stimulatory spots: Bespoke scripts and functions were written in MATLAB for the calculation of sampling efficiency and

protrusion index. For sampling efficiency of a cell, every pixel in the imaging field that is within the DIC boundary of the cell is marked as visited. For protrusion index, calculation of overlap with the stimulatory spot is the most important aspect, which is done by the AND operation between the segmented image of a cell (in DIC) and that of the stimulatory spot on which it resides. Segmentation of the DIC images of cells and spots was performed using in-built functions in TIAM. Only the arrested cells that do not have any 'neighbor', i.e. spots with a single arrested cell, are considered for the quantification of motile tendencies. This was done to avoid the technical ambiguity in delineating the boundaries of snugged cells and to avoid confounding of results due to influence of one arrested cell on the other.

All the reported statistical analyses were performed with Prism (Graphpad software).

Imaging of T-DC conjugates in collagen gels

Preparation of Monocyte derived DCs: Monocytes were isolated using RosetteSep Monocyte enrichment kit (StemCell #15068) as per the manufacturer's protocol. The monocytes were differentiated into DCs with 50μg/ml GM-CSF and 100μg/ml rhIL-4 (both from Peprotech) for 4 days in complete RPMI, then the following mixture of inflammatory cytokines was added for another 24hrs to induce maturation: 40ng/ml TNFα, 20ng/ml IFNγ, 10ng/ml IL1β (all from Peprotech) and 1μg/ml PGE2 (from Sigma).

Cell labelling: T-cells were labelled with 250 nM DeepRed Cell tracker (Invitrogen # C34565) for 20min at 37C in complete RPMI. The DCs were labelled similarly with 250 nM CMFDA (Invitrogen # C7025). The cells were used immediately for downstream experiments.

Loading of DCs with anti-CD3: Mature DCs were collected on day 5 after isolation and their concentration readjusted to 10^6/ml. Anti-CD3 (clone Okt3; 1 µg/ml) was added to the DCs and left to be capture by their Fc receptors for 30min at 37C. The DCs were then washed and used for stimulating T-cells.

3D collagen culture: Collagen master mix was prepared by mixing 75 µl of 3 mg/ml bovine collagen I (Cell Systems), with 10 µl 10xMEM and 5 µl of 7.5% sodium bicarbonate and CCL19 to a final concentration of (1 µg/ml). 200,000 T-cells and an equivalent number of DCs were mixed with the collagen master mix to reach a final concentration of 1.7mg/ml collagen. 50 µl of this collagen and cell mixture is introduced into a channel of VI Ibidi chamber and left to polymerise for 30-60mins at 37 ºC in an inverted position to prevent the cells from settling down on the coverslip. The wells feeding the channel were toped up with complete media and the sample is taken for 4D imaging.

Imaging: The collagen gel cell cultures were mounted on a PerkinElmer spinning disk fitted with CSU-10 head and a flash 4.0 sCMOS camera and enclosed in an environmental chamber (37 ºC and 5% CO2). Imaging was done using a 30x silicone oil objective from Olympus. Z-stacks were taken at 3 µm intervals and the time-lapse is recorded at 1 min^{-1}.

References

Celli, S., Garcia, Z., Beuneu, H., and Bousso, P. (2008). Decoding the dynamics of T cell-dendritic cell interactions in vivo. Immunological reviews *221*, 182-187.
Choudhuri, K., Llodra, J., Roth, E.W., Tsai, J., Gordo, S., Wucherpfennig, K.W., Kam, L.C., Stokes, D.L., and Dustin, M.L. (2014). Polarized release of T-cell-receptor-enriched microvesicles at the immunological synapse. Nature *507*, 118-123.
Dustin, M.L., Starr, T., Varma, R., and Thomas, V.K. (2007). Supported planar bilayers for study of the immunological synapse. Current protocols in immunology *Chapter 18*, Unit 18.13.
Mayya, V., Neiswanger, W., Medina, R., Wiggins, C.H., and Dustin, M.L. (2015). Integrative analysis of T cell motility from multi-channel microscopy data using TIAM. Journal of immunological methods *416*, 84-93.
Shen, K., Qi, J., and Kam, L.C. (2008a). Microcontact printing of proteins for cell biology. Journal of visualized experiments : JoVE.
Shen, K., Thomas, V.K., Dustin, M.L., and Kam, L.C. (2008b). Micropatterning of costimulatory ligands enhances CD4+ T cell function. Proceedings of the National Academy of Sciences of the United States of America *105*, 7791-7796.
Simson, R., Sheets, E.D., and Jacobson, K. (1995). Detection of temporary lateral confinement of membrane proteins using single-particle tracking analysis. Biophysical journal *69*, 989-993.
Wolf, I.M., Diercks, B.P., Gattkowski, E., Czarniak, F., Kempski, J., Werner, R., Schetelig, D., Mittrucker, H.W., Schumacher, V., von Osten, M.*, et al.* (2015). Frontrunners of T cell activation: Initial, localized Ca2+ signals mediated by NAADP and the type 1 ryanodine receptor. Science signaling *8*, ra102.
